# Multivariate Statistical Analysis of *Solidago canadensis* L. Essential Oil and Its Antifungal Mechanism Against Mulberry Sclerotinia Diseases

**DOI:** 10.3390/ijms27010049

**Published:** 2025-12-20

**Authors:** Jia-Xin Yang, Zhen-Zhen Lu, Sen Chen, Shi-Yi Lin, Xiao-Hui Yao, Tao Chen, Dong-Yang Zhang

**Affiliations:** 1 Jiangsu Key Laboratory of Sericultural and Animal Biotechnology, School of Biotechnology, Jiangsu University of Science and Technology, Zhenjiang 212100, China; 2 Key Laboratory of Silkworm and Mulberry Genetic Improvement, Ministry of Agriculture and Rural Affairs, Sericultural Scientific Research Center, Chinese Academy of Agricultural Sciences, Zhenjiang 212100, China

**Keywords:** *Ciboria shiraiana*, *Solidago canadensis* L., essential oil, antifungal mechanism, mulberry

## Abstract

*Ciboria shiraiana* (*C. shiraiana*), a pathogenic fungus, is a major threat to mulberry trees, causing mulberry sclerotinia diseases. Current control strategies primarily rely on chemical pesticides, whose long-term use leads to adverse effects such as pesticide residues, environmental pollution, and pathogen resistance. This study aimed to develop a green pesticide derived from the essential oil (EOs) of *Solidago canadensis* L. (*S*. *canadensis* L.) and to analyze its antifungal mechanism. SLEOs were extracted from flowers, leaves, and stems of *S*. *canadensis* L. via hydro-distillation. Their chemical composition was analyzed by GC-MS. Multivariate statistical analysis was used to assess compositional differences among SLEOs from various plant parts and evaluate the correlation between their chemical components and antifungal efficacy. The antifungal mechanism of SLEOs against *C. shiraiana* was investigated using an integrated approach combining transcriptomics with physiological and biochemical analyses. The EO yield varied with plant part: flowers yielded the most (1.00% ± 0.07%), followed by leaves (0.76% ± 0.04%) and stems (0.05% ± 0.01%). Flower EOs (FEOs) strongly inhibited *C. shiraiana*, with an EC_50_ value of 0.642 μL/mL. α-pinene and myrcene showed the highest correlation with antifungal activity. Transcriptomic and physiological data revealed that SLEOs compromise cell wall and membrane integrity, infiltrate cells, and trigger leakage of intracellular contents. Additionally, SLEOs inhibited activities of antioxidant enzymes (SOD, CAT, and POD), leading to intracellular ROS accumulation, oxidative stress, lipid peroxidation, and DNA damage. SLEOs constitute a promising natural and environmentally sustainable antifungal agent. Their activity is linked to specific components and a multi-target mechanism involving membrane disruption and oxidative stress induction. This study provides a foundation for developing plant-based agents to manage mulberry sclerotinia diseases.

## 1. Introduction

Plant pathogenic fungi pose persistent threats to worldwide crop production, causing substantial economic losses annually [1,2,3]. Mulberry (*Morus alba* L.) is an important economic crop whose fruits are rich in functional components such as anthocyanins and phenolic compounds, which possess antioxidant, antibacterial, and anti-inflammatory activities [4,5,6]. These attributes contribute to the widespread popularity of mulberry fruits among consumers. Mulberry sclerotinia diseases are highly destructive fungal infections that cause significant damage to its fruits [7]. *Ciboria shiraiana* (*C. shiraiana*) is a necrotrophic fungus that infects mulberry flowers, leading to hypertrophy sorosis scleroteniosis. It is the primary pathogen responsible for mulberry sclerotia diseases, causing severe annual losses in both the yield and quality of mulberry fruits [8]. Conventional control strategies rely heavily on synthetic chemical fungicides. However, the prolonged use of chemical pesticides results in adverse consequences, including pesticide residues, environmental pollution, and pathogen resistance [9,10,11,12]. Moreover, chemical fungicides have detrimental effects on non-target organisms and beneficial insects.

Botanical pesticides, derived from plant secondary metabolites, are gaining attention as promising alternatives to synthetic chemical pesticides. Botanical pesticides exhibit greater susceptibility to biological and photochemical degradation in the environment. They typically demonstrate lower persistence, reduced toxicity to non-target species, and a diminished propensity to induce resistance, thereby significantly mitigating environmental residues and long-term eco-risks [13]. Canadian goldenrod (*Solidago canadensis* L.) is a highly invasive plant that has rapidly spread across most Asian countries, central and western Europe, Australia, and New Zealand [14]. *S. canadensis* competes with native flora and disrupts ecological balance through allelopathic effects [15,16,17]. Despite its negative ecological impacts, its wide distribution, high biomass, easy accessibility, and low cost provide unique opportunities for resource utilization [18]. Previous studies have demonstrated that *S. canadensis* possesses antibacterial [19,20,21], anti-inflammatory [22], and antioxidant [23,24,25] activities.

Essential oils (EOs) are complex volatile mixtures derived from the secondary metabolites of aromatic plants, primarily composed of hydrophobic terpenoids and phenylpropanoids [26,27]. High volatility and lipophilic nature of EOs enable their rapid penetration into microbial cells, making them potential antifungal agents [28,29,30]. Furthermore, EOs are biodegradable and exert minimal effects on non-target species, not only slowing the development of resistance but also avoiding toxic effects on beneficial insects [31]. *S. canadensis* L. EOs (SLEOs) have been examined in various studies. Liu et al. [32] investigated the antifungal activity of SLEOs against *Botrytis cinerea* on strawberry fruits. The results indicated that the inhibitory effect of SLEOs on *B. cinerea* mycelial proliferation exhibited strong concentration dependency, with higher treatments causing progressively greater growth restriction. SLEO vapor at 0.1 mL/L maintained high sensory acceptability of strawberries while reducing gray mold incidence. Elshafie et al. [33] identified 32 constituents in SLEOs, with germacrene D, β-elemene, α-pinene, D-limonene, and bornyl acetate being the major ones. Previous studies have shown that SLEOs exhibit significant inhibitory activity against several plant pathogenic bacteria and postharvest plant pathogenic fungi. Therefore, SLEOs serve as potential natural fungicides.

However, no reports currently exist on the inhibition of *C. shiraiana* by SLEOs, and the antimicrobial components and mechanism of SLEOs require extensive investigation. Therefore, this study first identified the chemical composition of EOs extracted from different parts of *S. canadensis* L. using GC–MS. Multivariate statistical analyses, including principal component analysis (PCA), UpSet, partial least squares-discriminant analysis (PLS-DA), and correlation analysis, were employed to screen for the key components most strongly correlated with the antifungal efficacy of SLEOs. Finally, transcriptomic analysis was integrated with physiological and biochemical analyses to elucidate the antifungal mechanism against *C. shiraiana*. This research not only provides practical strategies for the sustainable management and efficient utilization of *S. canadensis* L. but also offers an effective control measure for mulberry sclerotia diseases.

## 2. Results and Discussion

### 2.1. EO Yield and Composition of Different Parts of S. canadensis L.

#### 2.1.1. SLEO Yields

*S. canadensis* L. has become widely invasive in southeastern China. In the sampling areas (Zhenjiang City), it exhibits extensive and expanding distribution, predominantly colonizing unmanaged herbaceous weed communities [34]. It has been reported that the plant height and several functional traits of *S. canadensis* L. increase significantly with the degree of invasion (Figure S1) [35]. In this study, the aboveground biomass of *S. canadensis* L. collected from twenty 1 m × 1 m quadrats averaged approximately 6.73 kg (FW) m^−2^. Leaves represent the dominant component of *S. canadensis* L., accounting for 59.05% ± 6.14% of the total plant dry weight. Previous studies indicate that the EO yields isolated from the dry matter of *S*. *canadensis* L. typically range from 0.21% to 0.34%. Flowers yield substantially higher EOs (0.35–1.47%) compared to other aerial parts (e.g., stems and leaves, yielding 0.11–0.16%) [36,37,38]. As shown in Table 1, SLEO yields in this study ranged from 0.05% to 1.00%, with the flower EOs exhibiting the highest yield at 1.00% ± 0.07%, markedly exceeding those from the leaf (0.76% ± 0.04%) and stem (0.05% ± 0.01%) fractions. Stems primarily contain low-volatility constituents associated with structural support, typically resulting in the lowest EO yields. Although flowers are not the principal biomass component, their elevated volatile oil yield designates them as the optimal raw material for SLEO extraction.

#### 2.1.2. SLEO Composition

**Figure 1:**
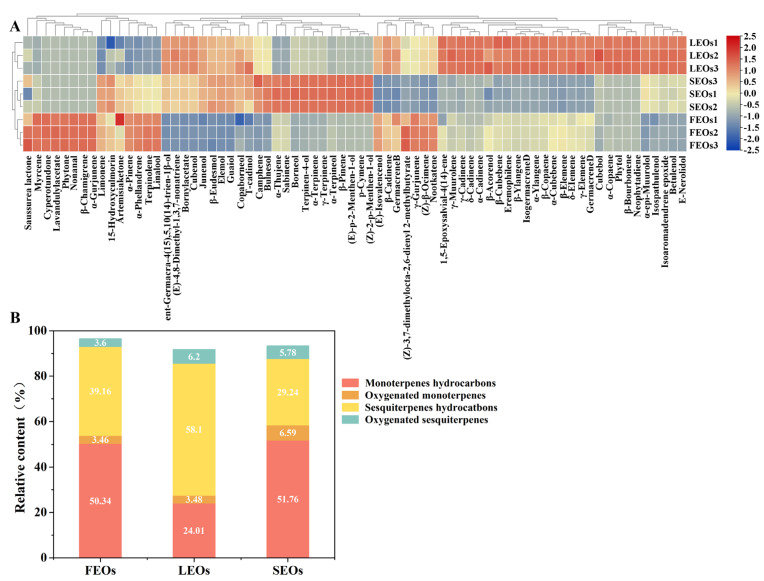
Chemical components and classification of SLEOs. (**A**) Chemical component heat map; (**B**) Classification of components. FEOs, SEOs, and LEOs represent the EOs derived from flowers, stems, and leaves of *Solidago canadensis* L., respectively.

Chemical constituents of SLEOs extracted from distinct botanical organs were analyzed by GC-MS. The total ion chromatograms are presented in Figure S2. Table S1 lists the 72 identified chemical constituents and their relative percentages in SLEOs extracted from flowers, leaves, and stems. As shown in Table S1 and Figure 1B, SLEOs were predominantly composed of monoterpenes (24.01–51.76%) and sesquiterpenes (29.24–58.10%), with β-pinene, α-pinene, limonene, sabinene, bornyl acetate, myrcene, β-ylangene, γ-muurolene, and germacrene D being the most abundant constituents (>5%). Among these, the sesquiterpene germacrene D (15.15–24.23%) was the most abundant component across all parts, reaching 24.23% in LEOs. The monoterpenes—α-pinene and limonene—were highly concentrated in FEOs (18.04% and 12.86%) and SEOs (13.82% and 13.55%) but significantly lower in LEOs (5.20% and 8.11%). Myrcene was most abundant in FEOs (9.66%), with significantly reduced levels in LEOs (2.97%) and SEOs (4.25%). Conversely, the sesquiterpenes β-ylangene (8.37%) and γ-muurolene (5.23%), along with the ester bornyl acetate (6.54%), peaked in LEOs and were substantially higher than in FEOs and SEOs. These compositional and quantitative differences among the SLEOs are further visualized in the heatmap in Figure 1A.

### 2.2. Multivariate Statistical Analyses of SLEOs

**Figure 2:**
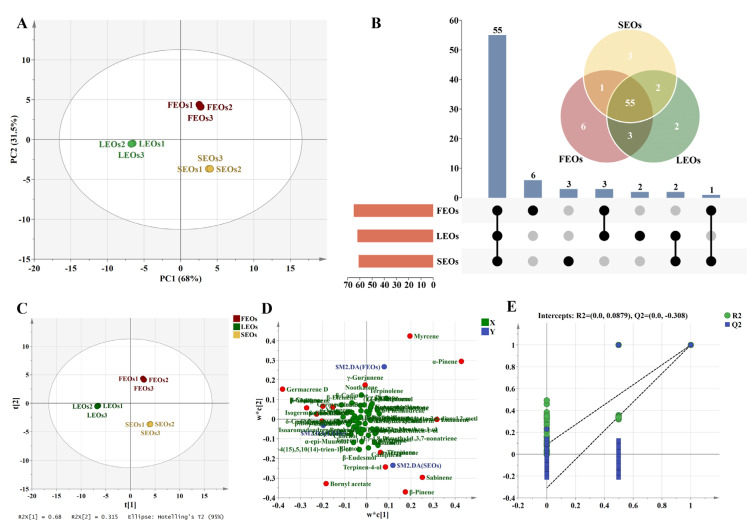
Multivariate statistical analyses of SLEOs. (**A**) PCA score map; (**B**) UpSet plot and Venn plot; (**C**) Score plot; (**D**) Loading plot; (**E**) Fitting curve of 200 permutation tests.

Multivariate statistical analyses were employed to examine the chemical composition of SLEOs derived from different plant parts. Initially, unsupervised PCA was conducted to visualize general clustering pattern [39]. The PCA score plot clearly shows the separation and grouping of different sample sets (Figure 2A). The first two principal components together explain 99.5% of the total variance in the original data, with contribution rates of 68% (PC1) and 31.50% (PC2). This indicates that these two components sufficiently capture the overall variation pattern and can be used to visually assess sample relationships. Specifically, the three biological replicates within each group cluster tightly on the plot, demonstrating high repeatability and consistency within groups. The three sample groups are distinctly separated, indicating significant compositional differences in the EOs from different plant parts. Figure 2B shows that SLEOs from the three parts shared 55 common components. Six components—phytone, α-gurjunene, cyperotundone, nonanal, lavandulyl acetate, and β-chamigrene—were unique to FEOs. Neophytadiene and phytol were exclusive to LEOs, while p-cymene, (E)-p-2-menthen-1-ol, and (Z)-2-p-menthen-1-ol were unique to SEOs.

**Figure 3:**
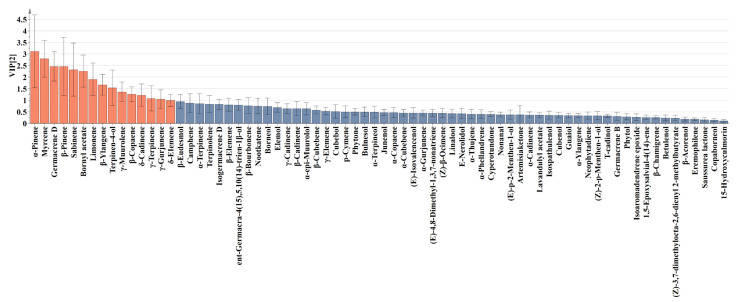
Variable importance in projection values derived from PLS-DA analysis.

PLS-DA was subsequently applied to further elucidate compositional differences in SLEO composition among different parts. This supervised multivariate technique establishes relationships between response and predictor variables through regression modeling, demonstrating superior predictive capability and clearer biological interpretation compared to conventional methods [40,41]. As shown in Figure 2C, the differences between SLEOs from different parts were distinct, similar to the PCA results (Figure 2A). Figure 2D illustrates the contribution of different components to SLEOs from various parts. FEOs showed positive correlations with monoterpene components such as myrcene and α-pinene. SEOs exhibited significant positive correlations with monoterpenes and oxygenated monoterpenes such as γ-terpinene, terpinen-4-ol, and β-pinene. In contrast, sesquiterpenes such as β-ylangene, γ-muurolene, and δ-cadinene, along with oxygenated sesquiterpenes, contributed predominantly to LEOs. Variable importance in projection (VIP) scores derived from the PLS-DA model were calculated to identify potential biomarkers and quantify their contribution to the model’s discrimination capability [42]. Model validation through 200 randomized permutation tests verified the predictive stability and model dependability of the PLS-DA (Figure 2E). The results showed R^2^ = (0.0, 0.0879) and Q^2^ = (0.0, −0.308). This Y-axis intercepts (R^2^Y < 0.3, Q^2^Y < 0.05) indicate that the model is not overfitted and is statistically reliable [42]. Figure 3 displays the VIP values of the 72 components. The top 15 components had high VIP values (≥1) and may contribute significantly to the variations in activity observed among the SLEOs. Additionally, α-pinene and β-Pinene, two naturally occurring isomers representative of the monoterpene class found in many plant EOs, have demonstrated inhibitory effects against both bacteria and fungi [43,44]. D-Limonene, commonly found in the peel EOs of citrus fruits, exhibits potent antifungal activity against *Aspergillus niger*, *Penicillium italicum*, *Botrytis cinerea*, and *Aspergillus flavus*, and has been developed as a botanical pesticide [45,46,47]. Numerous reports have confirmed the strong antimicrobial activity of germacrene D, γ-terpinene, and terpinen-4-ol [48,49]. Myrcene and sabinene have also been reported to exhibit synergistic effects in antifungal activity [50,51]. Overall, comprehensive, multidimensional statistical analysis provided valuable insights for screening compounds related to the antifungal activity of SLEOs.

### 2.3. Antifungal Activity of SLEOs

**Figure 4:**
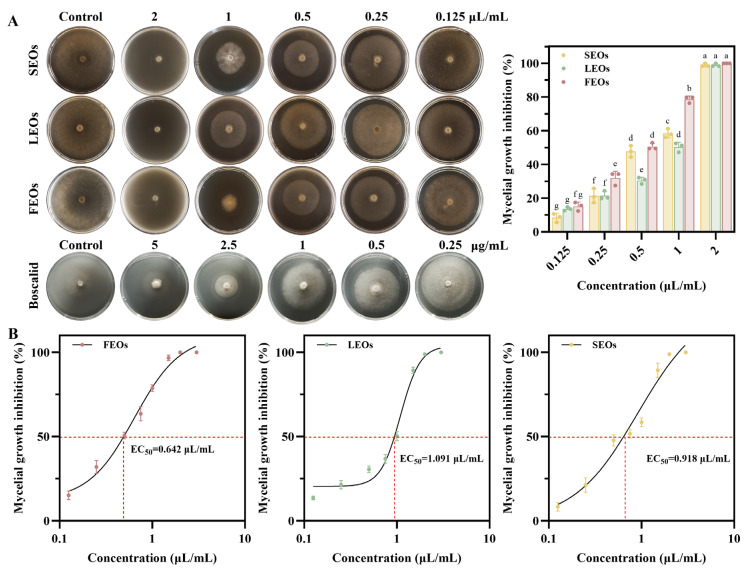
(**A**) Inhibitory activity of mycelial growth on PDA medium; (**B**) EC_50_ of FEOs, LEOs, and SEOs. Discs measured 6 mm in diameter, and values represent means ± standard deviations (*p <* 0.05). Means with different letters in a row are statistically significant (*p* < 0.05).

As shown in Figure 4, the antifungal activity of SLEOs against *C. shiraiana* was quantified using the mycelial growth rate method, and EC_50_ values of the three SLEOs were calculated [52]. The positive control showed a concentration-dependent antifungal effect, with an EC_50_ value of 3.380 μg/mL, which is consistent with previous reports (Figure S3) [53]. FEOs exhibited an EC_50_ value of 0.642 μL/mL (=0.552 mg/mL) against *C. shiraiana*, demonstrating superior antifungal efficacy compared to SEOs and LEOs, with this advantage becoming increasingly pronounced at higher concentrations. At a concentration of 2 μL/mL (=1.72 mg/mL), all three SLEOs achieved 100% mycelial growth inhibition. Botanical fungicides such as star anise oil, clove oil, and oregano oil have been extensively studied for their antifungal properties and have also shown inhibitory effects against *C. shiraiana* [54,55,56,57]. Huang et al. [56] investigated the antifungal efficacy of plant EOs against the pathogen causing mulberry sclerotinia diseases. Their results demonstrated that the minimum inhibitory concentrations (MICs) of star anise oil and clove oil against *C. shiraiana* were 0.25 mg/mL and 0.5 mg/mL, respectively. Liu et al. [57] compared the inhibitory effects of green, low-toxicity agents with conventional chemical fungicides on *C. shiraiana*. Green, low-toxicity agents such as carvacrol showed EC_50_ values ranging from 0.062 to 0.322 mg/mL, demonstrating comparable efficacy to the conventional fungicides and highlighting their potential for controlling mulberry sclerotinia diseases. SLEOs exhibited moderate antifungal activity against *C. shiraiana* but still demonstrated a clear concentration-dependent inhibitory effect. Given the abundant biomass, wide distribution, and easy accessibility of *Solidago canadensis* L., it holds substantial potential for development as a botanical fungicide to suppress *C. shiraiana*. Furthermore, the identification of EO components correlated with this antifungal activity can provide valuable insights for developing novel, safe, and efficient botanical fungicides targeting *C. shiraiana*.

**Figure 5:**
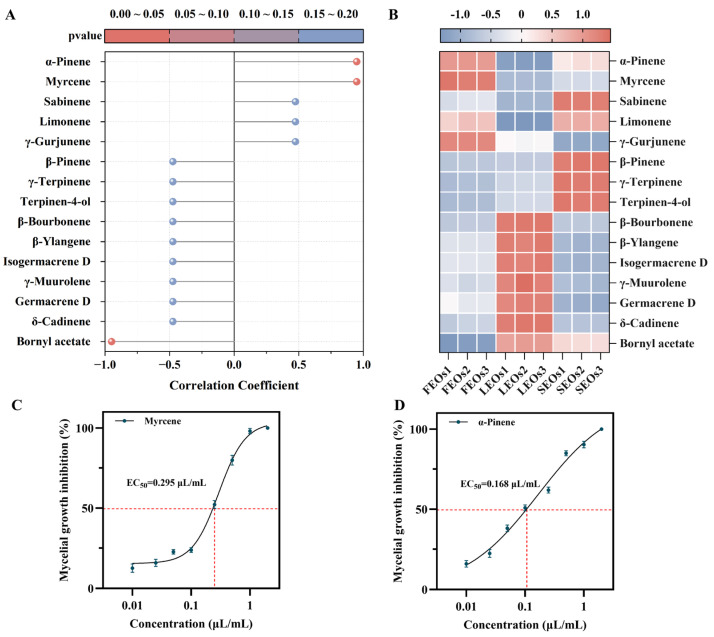
(**A**) Lollipop plot and (**B**) heatmap derived from Spearman correlation analysis between key components of SLEOs and antifungal properties; EC_50_ of (**C**) myrcene and (**D**) α-pinene.

Plant EOs are complex mixtures whose composition and relative abundance of constituents collectively influence their antifungal activity. The relationship between key SLEO components (VIP ≥ 1) and antifungal efficacy was thoroughly investigated using Spearman’s correlation analysis (Figure 5A), while a heatmap illustrated the distribution patterns of these 15 key components across the different SLEOs (Figure 5B). The results indicated that α-pinene and myrcene exhibited high correlation coefficients (close to 1) with the ability of SLEOs to inhibit *C. shiraiana*, alongside a statistically significant *p* < 0.05. Therefore, α-pinene and myrcene are likely the components contributing most significantly to the antifungal activity of SLEOs. As shown in Figure 5C,D and Figure S4, α-pinene (0.168 μL/mL) and myrcene (0.295 μL/mL) exhibit significantly lower EC_50_ values compared to the SLEOs. At a concentration of 1 μL/mL, both compounds suppressed mycelial growth by more than 90%. This superior antifungal potency, exceeding that of the SLEOs themselves, provides further validation that α-pinene and myrcene are the key components most responsible for the antifungal activity. Previous studies have shown that α-pinene can inhibit ergosterol synthesis and exerts destructive effects on fungal cell membranes [43,44]. Consequently, the antifungal mechanism of SLEOs against *C. shiraiana* may primarily target the cell membrane. The identification of key components significantly positively correlated with the antifungal activity of SLEOs, based on Spearman’s correlation analysis, provides a critical reference for elucidating the antifungal mechanism of SLEOs against *C. shiraiana*. The superior yield and antifungal efficacy of FEOs, compared to SEOs and LEOs, highlight their greater potential as a plant-based antimicrobial agent. Consequently, FEOs were chosen for the antifungal mechanism studies that followed.

### 2.4. Antifungal Mechanism of FEOs Based on Transcriptomics

#### 2.4.1. Transcriptomics

To investigate the mechanism of FEOs against *C. shiraiana*, transcriptome sequencing analysis was conducted on FEOS-treated *C. shiraiana*. Herein, genes with a fold change ≥ 1 and *p*-adj ≤ 0.05 were designated as DEGs. As shown in the volcano plot (Figure S5), 3289 DEGs were identified between the FEOs-treated and control groups, including 1864 upregulated and 1425 downregulated genes.

**Figure 6:**
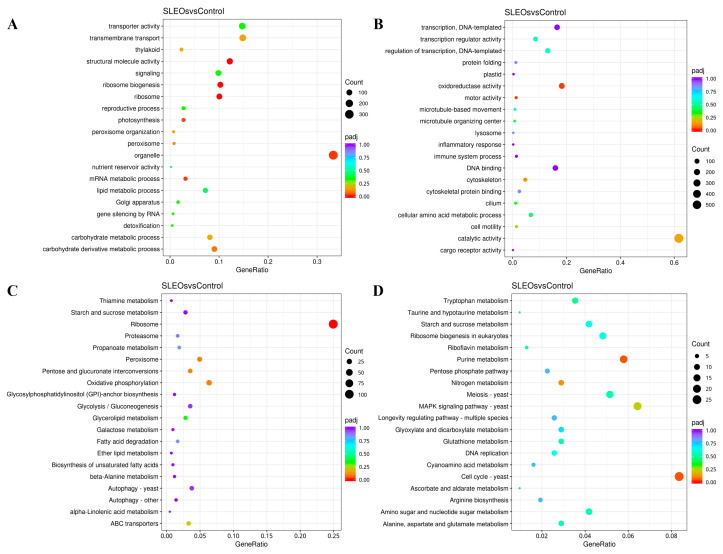
GO enrichment analysis of (**A**) upregulated and (**B**) downregulated DEGs in *C. shiraiana*. KEGG enrichment analysis of (**C**) upregulated and (**D**) downregulated DEGs in *C. shiraiana*.

Based on Gene Ontology (GO) annotation, DEGs were functionally classified. As illustrated in Figure S6, the GO analysis revealed distinct functional patterns: under Biological Processes, differentially expressed genes showed significant enrichment in cellular and metabolic processes. In cellular components, DEGs primarily comprised protein-containing complexes and cellular anatomical entities. In the Molecular Function category, DEGs were predominantly associated with catalytic activity and binding. The enrichment of DEGs in metabolic processes and catalytic activity suggests that FEOs may interfere with the metabolic pathways or enzymatic functions of *C. shiraiana*. Figure 6A,B display the top 20 bubble charts for GO enrichment of the upregulated and downregulated DEGs, respectively. The downregulation of terms, including microtubule organizing center, microtubule-based movement, cytoskeleton, cilium, and cell motility, indicates that FEOs may disrupt the microtubule/microfilament system, inhibit fungal cell mitosis, and suppress hyphal tip growth [58,59]. The inhibition of transcription function suggests that FEOs may suppress gene expression, impairing protein synthesis required for stress response and essential life activities and hindering the gene expression essential for maintaining basic life activities [60,61,62]. The downregulation of oxidoreductase activity indicates that FEOs may disrupt the oxidative phosphorylation respiratory chain, leading to a severe deficiency in ATP production. Conversely, the significant enrichment of catalytic activity and cellular amino acid metabolic process suggests inhibition of key synthesis and catabolism, implying that FEOS treatment may place *C. shiraiana* cells in a “starvation” state [53,63]. Enrichment in lipid metabolism suggests potential damage to membrane structure and also reveals a significant impact on fungal transport activity and transmembrane transport, indicating that FEOs may severely disrupt the structure and function of the fungal cell membrane [61,62]. As observed in Figure S7, DEGs were significantly enriched in catalytic activity and cellular amino acid metabolic process, indicating that FEOs may cause cell wall stress in fungi [60,61,62].

Changes in functional pathways of *C. shiraiana* following FEOS treatment were further investigated through KEGG enrichment analysis. The top 20 most significant pathways were visualized using bubble plots (Figure S8 and Figure 6C,D). Pathways enriched in FEOS-treated samples were primarily associated with the cell wall, cell membrane, and cell proliferation and growth. Specifically, enrichment in pathways, including amino sugar and nucleotide sugar metabolism, glycerolipid metabolism, GPI-anchor biosynthesis, galactose metabolism, ether lipid metabolism, and biosynthesis of unsaturated fatty acids, indicates that FEOs directly impair the structural integrity of the cell wall while simultaneously disturbing membrane architecture and functionality.

**Figure 7:**
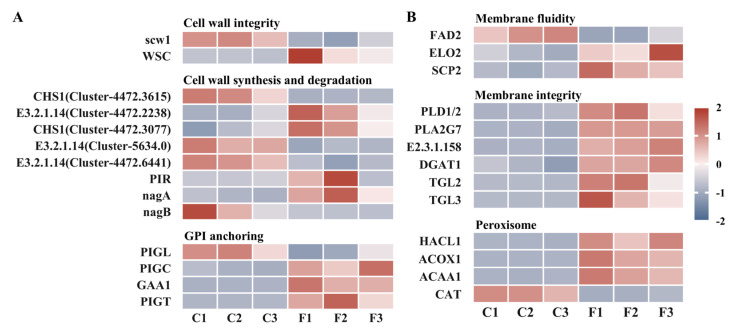
Heatmap of (**A**) cell wall-related DEGs and (**B**) cell membrane- and peroxidase-related DEGs.

GO and KEGG analyses indicated that the antifungal effect of FEOs on *C. shiraiana* primarily targeted the cell wall, cell membrane, and antioxidant system. Based on these findings, DEGs were further classified based on gene function, and their expression was visualized using a heatmap. As shown in Figure 7A, the expression of the *scw1* gene, encoding a cell wall integrity protein, was significantly downregulated. Concurrently, WSC proteins (cell wall integrity and stress response component) were upregulated, activating the MAPK pathway in an attempt to repair cell wall damage [64,65]. Chitin is a crucial component of the fungal cell wall; a reduction in chitin content affects cell wall integrity and inhibits fungal growth [66]. Following treatment with FEOs, the bidirectional regulation of chitinases and chitin synthases reflects the complex repair and compensatory mechanisms employed by *C. shiraiana* to maintain cell wall homeostasis under FEOS stress. Furthermore, as shown in Figure 7B, significant downregulation of *FAD2* genes reduced unsaturated fatty acid content, while upregulation of *ELO2* gene expression catalyzed the synthesis of long-chain fatty acids. This decreased membrane fluidity and increased rigidity and brittleness, impairing environmental adaptability [67,68,69,70]. Ergosterol serves as a fundamental structural element in fungal membranes, playing a crucial role in preserving membrane flexibility, structural stability, and biological functions. The upregulation of *SCP2* suggests that the fungus may maintain and repair membrane structure and function through sterol transport [43,61]. Significant upregulation of hydrolytic enzymes such as PLA2G7, PLD1/2, and TGL further indicates compromised membrane integrity. ACOX1 and ACAA1, enzymes catalyzing the first and last steps of β-oxidation, respectively, may synergistically promote membrane lipid degradation. Notably, ACOX1 and HACL1 can cooperatively catalyze the dehydrogenation of long-chain fatty acyl-CoAs to produce H_2_O_2_. Simultaneously, the significant downregulation of CAT indicated that FEOs inhibited fungal antioxidant enzymes, leading to ROS accumulation and potential membrane lipid peroxidation [71].

#### 2.4.2. Effects of FEOs on Morphology and Ultrastructure of *C. shiraiana*

**Figure 8:**
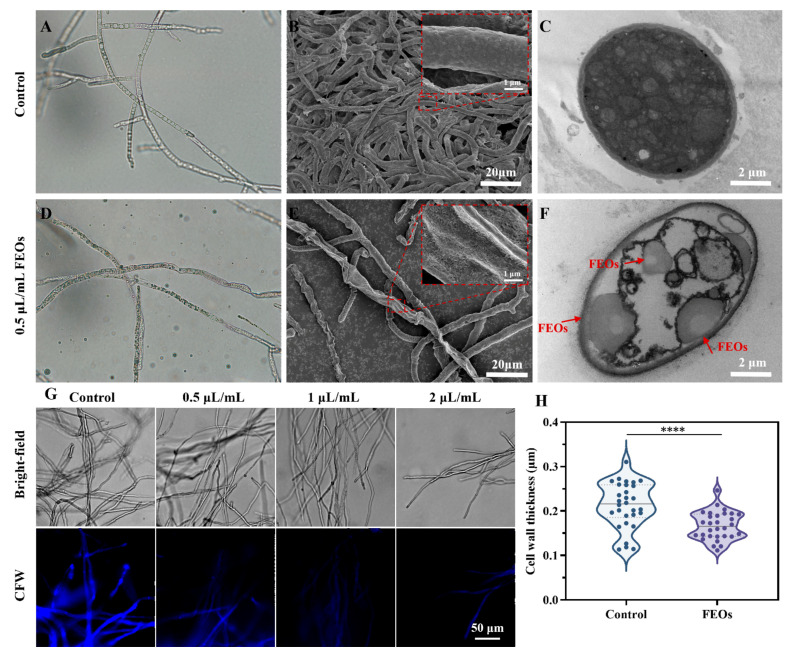
(**A**,**D**) Optical microscopy images, ×40, (**B**,**E**) SEM images and (**C**,**F**) TEM images of *C. shiraiana*; (**G**) CWS staining of *C. shiraiana*; (**H**) Influence on cell wall thickness. FEOs: flower essential oil. **** *p* < 0.0001.

RNA-seq data demonstrated that the antifungal mechanism of FEOs against *C. shiraiana* mainly involves compromising the integrity and functionality of both cell wall and membrane systems. Accordingly, this section investigates the morphological alterations induced by the EOs in *C. shiraiana* (Figure 8). Optical microscopy (OM) and SEM revealed that the mycelia in the untreated control group had a smooth surface and regular shape (Figure 8A,B). In contrast, FEOS treatment induced pronounced morphological alterations in hyphae, including severe shrinkage, surface collapse, and the emergence of numerous pores (Figure 8D,E). TEM was used to observe the impact of FEOs on the cellular ultrastructure of *C. shiraiana*. Hyphal cells in the control group displayed an intact cell wall and membrane structure, uniformly distributed cytoplasm, and abundant organelles (Figure 8C). Conversely, FEOS-treated hyphal cells exhibited a thinner cell wall and evident membrane rupture. Most intracellular organelles were diminished or dissolved, appearing indistinct, and numerous vacuoles were observed within the cells. Additionally, penetration of EO components through the cell wall and membrane into the intracellular space was observed (Figure 8F).

#### 2.4.3. Effects of FEOs on Cell Wall of *C. shiraiana*

As shown in Figure 8G, the untreated *C. shiraiana* exhibited uniformly distributed blue fluorescence, with clearly visible chitin septa. Following FEOS treatment, fluorescence intensity in fungal hyphae progressively declined with increasing FEOS concentrations, and the chitin septa within the hyphae gradually disappeared. Compared to the control group, fungal cell walls treated with FEOs were markedly thinner (Figure 8H). These observations indicate that FEOs significantly reduce the chitin content in the cell wall of *C. shiraiana*, thereby compromising cell wall integrity. This outcome aligns with the transcriptome analysis results.

#### 2.4.4. Effects of FEOs on Cell Membrane of *C. shiraiana*

**Figure 9:**
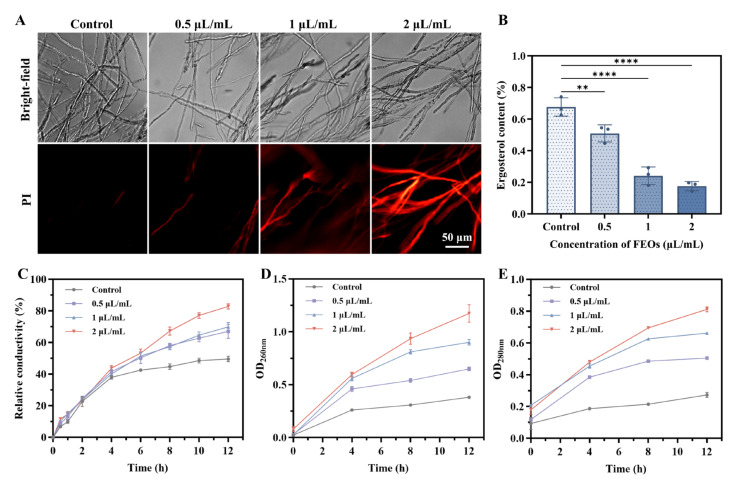
(**A**) PI staining of *C. shiraiana*; Effects on (**B**) ergosterol content, (**C**) relative conductivity value, and (**D**,**E**) cellular content leakage. The data are shown as mean ± standard deviation (SD), n = 3, error bars are SDs. ** *p* < 0.01, and **** *p* < 0.0001.

Membrane integrity was evaluated using PI staining. PI cannot penetrate the intact membranes of viable cells but can enter compromised cells to bind DNA, emitting red fluorescence. As shown in Figure 9A, PI fluorescence was minimal in the control group, whereas treatment with 0.5 μL/mL FEOs induced sparse fluorescence without notable membrane disruption. Higher FEOS concentrations markedly increased fluorescence intensity and spatial distribution, indicating severe membrane integrity loss and confirming the disruptive effects of FEOs on *C. shiraiana* membranes.

Ergosterol serves as a core structural and functional molecule in fungal cell membranes. Embedded within the phospholipid bilayer, its rigid sterol ring and hydrophobic side chain interact with fatty acid chains of phospholipids, playing a vital role in maintaining membrane stability, fluidity, and permeability [43,61]. As shown in Figure 9B, treatment with 0.5 μL/mL FEOs significantly reduced ergosterol content compared to the control (*p* < 0.01), with progressively greater inhibition observed at higher FEOs concentrations. These results indicate that FEOs disrupt fungal cell membranes by inhibiting ergosterol biosynthesis.

Membrane permeability was assessed via relative electrolyte leakage, wherein elevated conductivity reflects increased membrane damage. Extracellular conductivity increased sharply within 0.5 h and increased progressively with time and FEOS concentrations (Figure 9C). Treated mycelia exhibited significantly higher conductivity than controls. Initial membrane compromise permits leakage of small molecules, followed by macromolecules such as nucleic acids and proteins [67,70]. To further validate membrane disruption, cellular content leakage was quantified. As shown in Figure 9D,E, absorbance at OD_260nm_ (nucleic acids) and OD_280nm_ (proteins) increased significantly with FEOS concentrations and exposure time, demonstrating progressive leakage of biomacromolecules into the extracellular matrix. Collectively, these results demonstrate that FEOs induce membrane lipid peroxidation, structural disruption, and increased permeability, culminating in cellular content leakage and functional collapse.

#### 2.4.5. Effects of FEOs on Endogenous ROS and Oxidative Damage of *C. shiraiana*

**Figure 10:**
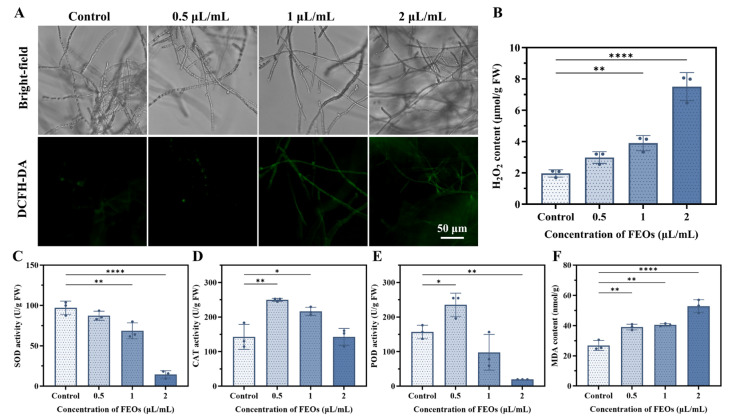
(**A**) DCFH-DA staining of *C. shiraiana*; (**B**) H_2_O_2_, (**C**) SOD, (**D**) CAT, (**E**) POD, and (**F**) MAD activities in *C. shiraiana*. The data are shown as mean ± standard deviation (SD), n = 3, error bars are standard deviations. * *p* < 0.05, ** *p* < 0.01, and **** *p* < 0.0001.

ROS accumulation can damage biological macromolecules, leading to lipid peroxidation, DNA damage, protein oxidation, membrane disruption, enzyme inactivation, and cell death [72,73]. Herein, intracellular ROS levels were evaluated using DCFH-DA staining. DCFH-DA itself is non-fluorescent but is hydrolyzed by intracellular esterases and subsequently oxidized by ROS to form fluorescent DCF. As shown in Figure 10A, the fluorescence intensity was low in the control group but markedly increased in FEOs-treated fungal hyphae in a concentration-dependent manner.

H_2_O_2_ is the predominant ROS in biological systems and oxidizes macromolecules such as nucleic acids and proteins. Once H_2_O_2_ levels exceed the scavenging capacity of the fungal antioxidant system, it causes oxidative damage to fungal cells and membrane disruption, inhibiting fungal growth or causing cell death [74,75]. As shown in Figure 10B, H_2_O_2_ content significantly increased with rising FEOS concentrations, indicating that FEOs elevate intracellular H_2_O_2_ levels in *C. shiraiana* beyond its antioxidant capacity, resulting in oxidative damage.

SOD, POD, and CAT constitute crucial antioxidant enzyme systems in fungi, scavenging intracellular ROS and protecting against oxidative damage [74]. SOD serves as the first line of defense, catalyzing the dismutation of harmful superoxide anion radicals (O_2_^−^) into H_2_O_2_ and O_2_, while CAT and POD further decompose H_2_O_2_. As shown in Figure 10C, FEOS treatment significantly suppressed SOD activity in *C. shiraiana*. Fungicides can induce oxidative stress in fungal cells, triggering increased antioxidant enzyme activity to mitigate ROS-induced damage [74]. At an FEOS concentration of 0.5 μL/mL, CAT and POD activities were elevated; however, further increases in FEOS concentrations led to progressive enzymatic decline (Figure 10D,E). These results demonstrate that low concentrations of FEOs induce oxidative stress in *C. shiraiana*, upregulating CAT and POD activities to eliminate excess ROS. Increased FEOS concentrations disrupt the fungal antioxidant enzyme system, causing intracellular ROS accumulation and oxidative damage.

MDA, a terminal product of cellular peroxidation, is generated during membrane lipid peroxidation under adverse conditions. MDA content serves as an indicator of cell membrane damage severity [74]. As shown in Figure 10F, MDA content in FEOs-treated *C. shiraiana* hyphae was significantly higher than in the control group and increased progressively with FEOS concentrations. This indicates that FEOs induce lipid peroxidation in the fungus, causing damage to the cell membrane.

DNA, the central carrier of fungal genetic information and vital cellular functions, is susceptible to oxidative damage. Such damage disrupts gene expression, leading to cell cycle arrest or apoptosis [76,77]. To assess DNA integrity, fungal hyphae were stained with DAPI. As shown in Figure S9, FEOs-treated *C. shiraiana* hyphae exhibited intensified and condensed blue fluorescence relative to the control group. These observations indicate that FEOS treatment induces DNA damage in *C. shiraiana*, compromising fungal reproduction and genetic stability.

**Figure 11:**
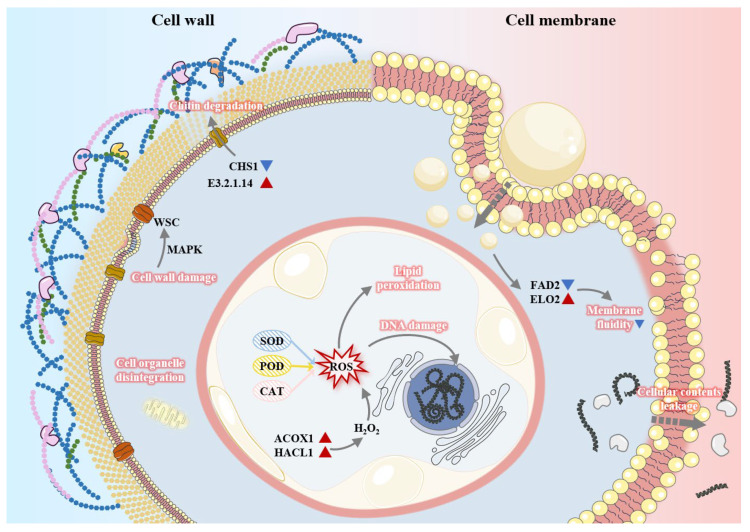
Schematic illustration of *C. shiraiana* after treatment with FEOs. *CHS1, E3.2.1.14, WSC, FAD2,* and *ELO2* are the gene names of *C. shiraiana*.

Figure 11 illustrates the proposed mechanism of action of FEOs against *C. shiraiana*, derived from integrated transcriptomic sequencing and analysis. First, FEOs induce degradation of chitin and thinning of the cell wall by bidirectionally regulating *CHS1* and *E3.2.1.14*, thereby compromising cell wall integrity. Concurrently, FEOs inflict irreversible damage on the cell membrane by disrupting its fluidity and structural integrity. Subsequently, FEOs penetrate through the damaged cell wall and membrane to enter the cell, while large molecules within the cell (such as nucleic acids and proteins) leak out. Furthermore, FEOs inhibit the activities of key antioxidant enzymes, including SOD, POD, and CAT, resulting in the dysfunction of the antioxidant enzyme system. This enzymatic disruption promotes intracellular ROS accumulation and oxidative stress, initiates membrane lipid peroxidation, and induces DNA damage in *C. shiraiana*, thereby impairing its reproductive capacity and genetic stability.

## 3. Materials and Methods

### 3.1. Materials and Chemicals

In September 2024, aboveground plant samples (flowers, stems, and leaves) of *S. canadensis* L. at full bloom were randomly collected in Zhenjiang City, Jiangsu Province, China (32.10° N, 119.60° E). Then the leaves, flowers and stems were separated, and they were air-dried at room temperature in the dark.

The *C. shiraiana* strain used was gifted by the National Key Laboratory of Silkworm Genome Biology, Southwest University. The strains were cultivated on potato dextrose agar (PDA) and kept at 28 ± 1 °C for mycelial development.

### 3.2. Extraction of SLEOs

Briefly, 100 g of dried *S. canadensis* L. sample was subjected to hydrodistillation with 1000 mL of distilled water using a Clevenger-type apparatus for 2 h to obtain SLEOs. The collected SLEOs were subsequently dried over Na_2_SO_4_ and then stored at 4 °C [42]. The SLEO yields were estimated on a dry-weight basis (*w*/*w*).

### 3.3. Chromatographic Analysis

The method was adapted from a reported procedure in the literature with modifications. SLEOs were diluted in n-hexane at 1:1000 (*v*/*v*) dilution. Analysis was performed using a 7890B-5977B GC–MS (Agilent Technologies, Santa Clara, CA, USA) equipped with an HP-5MS capillary column (30 m × 250 μm × 0.25 μm; Agilent Technologies, Santa Clara, CA, USA). The injection volume was 2 μL in splitless mode. Both the injector and transfer line were maintained at 280 °C, with high-purity helium serving as carrier gas at 1.0 mL/min constant flow. The oven temperature program included: 5 min at 60 °C, ramp to 220 °C at 4 °C/min, subsequent increase to 280 °C at 11 °C/min (15 min hold), and finally increased to 300 °C at 11 °C/min (5 min hold) [78].

Mass spectrometric detection was conducted using electron impact ionization at 70 eV with the ion source maintained at 200 °C. Full-scan data collection was performed across the *m*/*z* 29–650 range. The obtained mass spectra were compared with those reported in the literature [33,79], and the fragmentation patterns of the mass spectra and their retention indices (RIs) were compared with the NIST20 library. The relative content of each component was calculated based on its relative peak area percentage.

### 3.4. Antifungal Activity of Different Plant Part EOs of S. canadensis L. In Vitro

The in vitro antifungal efficacy of SLEOs was evaluated using the mycelial growth rate method [52]. PDA plates were treated with different concentrations of flower EOs (FEOs), leaf EOs (LEOs), and stem EOs (SEOs), with 0.1% Tween-20 (*v*/*v*) serving as the control group. Boscalid was used as a positive control. Mycelial plugs (6 mm diameter) were inoculated onto the treated PDA plates and cultured at 28 ± 1 °C for 3 days. All tests were performed in triplicate. The in vitro antifungal activity was quantified by determining the half-maximal effective concentration (EC_50_), while the inhibition rate was calculated using the following formula:

(1) $$Mycelial\;growth\;inhibition(\%)\;=\;[(d_{c}-d_{t})/(d_{c}-6\;mm)]\times 100$$

where d*_c_* and d*_t_* represent average colony diameters of the control and treatment groups, respectively.

### 3.5. Transcriptomic Analysis

Mycelial discs were transferred to 60 mL of potato dextrose broth (PDB) for liquid cultivation and incubated with shaking at 150 rpm at 28 ± 1 °C for three days. After treatment with 0.1% Tween-20 (control) or 0.5 μL/mL FEOs for 24 h, the mycelia were vacuum-filtered and washed thrice with 0.01 M phosphate-buffered saline (PBS, pH 7.2–7.4). Finally, the blot-dried mycelia were flash-frozen in liquid nitrogen.

High-throughput RNA-seq analysis was performed by Novogene Bioinformatics Technology Co., Ltd. (Beijing, China) utilizing the Illumina NovaSeq 6000 platform (Illumina, San Diego, CA, USA). Sequencing produced 150-bp paired-end sequencing libraries, yielding a minimum of 6 GB high-quality data per biological replicate. Raw sequencing data were subjected to quality control using Fastp (v0.23.2) to remove adapters and low-quality reads (Q-score < 20). De novo transcriptome assembly was executed with Trinity (v2.15.1), followed by functional annotation against GO and KEGG databases. Differentially expressed genes (DEGs) were classified using DESeq2 (v1.40.2) (|log2FC| > 1, *p*-adj < 0.05).

### 3.6. Observation of Mycelium Morphology and Ultrastructure

The assays were conducted according to previously reported methods [54]. Mycelial discs were transferred to 60 mL of PDB for liquid cultivation and incubated with shaking at 150 rpm at 28 ± 1 °C for three days. Subsequent morphological examination of hyphae was conducted using bright-field microscopy (Model BX51, OLYMPUS Co., Ltd., Tokyo, Japan).

Scanning electron microscopy (SEM) was performed following established procedures [54]. Mycelia treated with 0.5 μL/mL FEOs for 24 h were fixed in 2.5% glutaraldehyde for 4 h, followed by three 15-min washes with 0.01 M PBS. Samples were then dehydrated through a graded ethanol/water series. After freeze-drying, specimens were gold-sputtered using an ion sputter coater and examined using SEM (Model SU8600, Hitachi Co., Ltd., Tokyo, Japan).

Transmission electron microscopy (TEM) was carried out as described in [53]. Ultrastructural features of *C. shiraiana* were observed using TEM (Model HT-7800, Hitachi Co., Ltd., Tokyo, Japan).

### 3.7. Effect on Cell Wall of C. shiraiana

A small amount of mycelium treated with FEOs was placed on a glass slide, washed with 0.1 M PBS, stained by adding 10 μL of calcofluor white stain (CWS) dye and a 10% (*w*/*v*) KOH solution onto the mycelial surface, mounted with a cover slip, and observed under a fluorescence microscope (CKX31, OLYMPUS, Co., Ltd., Tokyo, Japan).

### 3.8. Effect on ROS and Oxidoreductase Activity of C. shiraiana

The ROS level was determined according to a previously reported method [53]. A small amount of mycelium treated with FEOs was washed three times with 0.1 M PBS and subsequently incubated with 10 μM DCFH-DA fluorescent probe for 20 min at 37 °C under dark conditions. After repeated PBS washing, the mycelium was subjected to fluorescence microscopic examination and image acquisition (CKX31, OLYMPUS, Co., Ltd., Tokyo, Japan).

The activities of antioxidant enzymes were measured using commercial assay kits (Beijing Solarbio Technology Co., Ltd., Beijing, China). Mycelium (100 mg) treated with different concentrations of FEOs was homogenized in 1 mL of extraction buffer in an ice bath. The contents of MDA and H_2_O_2_, as well as the activities of SOD, CAT, and POD were determined according to the manufacturer’s instructions.

### 3.9. Effect on Cell Membrane of C. shiraiana

Ergosterol content was determined according to the reported method [43]. The mycelial samples were incubated with 5 mL of 25% (*w*/*v*) KOH-ethanol solution in a water bath at 85 °C for 1 h. After adding 2 mL of sterile water and 5 mL of n-hexane, the mixture was vortexed and centrifuged. The absorbance of the supernatant was measured at 282 nm and 230 nm using a UV–vis spectrophotometer (LAMBDA 25, PerkinElmer Inc., Waltham, MA, USA).

The integrity of fungal cell membranes was evaluated through propidium iodide (PI) staining. Mycelial samples treated with different concentrations of FEOs were washed with 0.1 M PBS in triplicate. The mycelia were incubated in a 20 μg/mL PI staining solution for 20 min at room temperature and subsequently washed with 0.1 M PBS. Finally, the processed samples were examined and imaged using a fluorescence microscope (CKX31, OLYMPUS, Co., Ltd., Tokyo, Japan).

The effect of FEOs treatment on the membrane permeability of *C. shiraiana* was evaluated by measuring relative electrical conductivity [80]. Mycelia were added to PDB medium and cultured at 28 ± 1 °C for 3 d. The collected mycelia (400 mg) were added to 40 mL of sterile distilled water containing different concentrations of FEOs, with 0.1% Tween-20 (*v*/*v*) serving as a blank control. The conductivity of the supernatant was measured using a conductivity meter at 0, 0.5, 1, 2, 4, 6, 8, 10, and 12 h (the value at 0 h was denoted as L_0_, and values from 0.5–12 h were denoted as L_1_) (FE30, Mettler-Toledo GmbH, Switzerland). Following 30 min of boiling and subsequent cooling to ambient temperature, the electrical conductivity (designated as L_2_) of the solutions was measured. The relative conductivity was determined using the following formula:

(2) $$Relative\;electric\;conductivity=[(L_{1}-L_{0})/(L_{2}-L_{0})]\times 100$$

The release of intracellular materials was measured with modifications according to the method of Zhao et al. [80]. Mycelia cultured in PDB medium for 3 d (400 mg) were added to 40 mL of sterile distilled water containing different concentrations of FEOs, with 0.1% Tween-20 (*v*/*v*) as a blank control. After incubation at 28 ± 1 °C with shaking at 150 rpm, the absorbance of the supernatant at OD_260_ and OD_280_ was measured using a UV–Vis spectrophotometer (LAMBDA 25, PerkinElmer Inc., Waltham, MA, USA) at 0, 4, 8, and 12 h.

### 3.10. Effect on DNA Damage of C. shiraiana

The extent of DNA damage was assessed using 4′,6-Diamidino-2-phenylindole dihydrochloride (DAPI) staining. Mycelial samples treated with different concentrations of FEOs were washed three times with 0.1 M PBS. Specimens were fixed with 70% ethanol (*v*/*v*) for 30 min. Subsequently, samples were immersed in 5 μg/mL DAPI staining solution and incubated at room temperature for 20 min, followed by three washes with 0.1 M PBS. DAPI-stained samples were observed and photographed under a fluorescence microscope (CKX31, OLYMPUS, Co., Ltd., Tokyo, Japan).

### 3.11. Statistical Analyses

Statistical analyses were carried out with SPSS software (version 23.0; IBM, Endicott, NY, USA) for one-way ANOVA and Spearman’s rank-order correlation methods. Multivariate analyses including PCA and PLS-DA were executed in SIMCA (Version 14.1; Umetrics, Umeå, Sweden). Figures were plotted using Origin (OriginPro, Version 2024. OriginLab Corporation, Northampton, MA, USA). Three independent experiments were conducted for all assays, with quantitative data expressed as means ± standard deviation.

## 4. Conclusions

In summary, 72 components of SLEOs were identified, and compositional and antifungal activity differences among plant parts were analyzed for the first time. FEOs demonstrated the most potent antifungal efficacy against *C. shiraiana*, showing an EC_50_ of 0.642 μL/mL. Among them, α-pinene and myrcene showed the strongest correlation with antifungal activity. The mechanism of action of SLEOs against *C. shiraiana* was further elucidated using transcriptomic analysis. The results indicated that SLEOs inhibit the synthesis of chitin and ergosterol, damage the cell wall and membrane, and enter the cell to exert their effects. SLEOs also induce intracellular ROS accumulation by modulating the activities of SOD, CAT, and POD enzymes, elevate levels of the lipid peroxidation product MDA, and damages the membrane functionality, resulting in the leakage of vital cellular constituents. Additionally, SLEOs cause DNA damage. Our findings demonstrate the potential of *S. canadensis* L. as a viable and sustainable source of EOs, offering an eco-friendly alternative for controlling mulberry sclerotinia disease.

## Figures and Tables

**Table 1:** Biomass characteristics of different parts of *S*. *canadensis* L.

	Flower	Leaf	Stem
Moisture content (wt%)	64.64 ± 1.46 ^a^	62.08 ± 1.30 ^b^	52.11 ± 1.53 ^c^
Dry weight fraction (%)	19.53 ± 2.99 ^b^	59.05 ± 6.14 ^a^	21.43 ± 4.68 ^b^
Biomass (kg (DW)/m^2^)	0.64 ± 0.16 ^b^	1.95 ± 0.43 ^a^	0.72 ± 0.26 ^b^
Yields of SLEO (%DW)	1.00 ± 0.07 ^a^	0.76 ± 0.04 ^b^	0.05 ± 0.01 ^c^
Density of SLEOs (g/mL)	0.86 ± 0.00	0.86 ± 0.00	0.86 ± 0.00

Notes: Means with different letters in a row are statistically significant (*p* < 0.05).

## Data Availability

The original contributions presented in this study are included in the article/Supplementary Material. Further inquiries can be directed to the corresponding authors.

## Supplementary Materials

The following supporting information can be downloaded at: https://www.mdpi.com/article/10.3390/ijms27010049/s1.

## References

[1] Stukenbrock E., Gurr S. (2023). Address the growing urgency of fungal disease in crops. Nature.

[2] White J.C., Gardea-Torresdey J. (2018). Achieving food security through the very small. Nat. Nanotechnol..

[3] Li P.F., Tedersoo L., Crowther T.W., Wang B.Z., Shi Y., Kuang L., Li T., Wu M., Liu M., Luan L. (2023). Global diversity and biogeography of potential phytopathogenic fungi in a changing world. Nat. Commun..

[4] Zhang L., Chen Q., Chen Z., He T., Yu M., Zhang Y., Nan H., Huang Q., Zhao T. (2024). Anti-skin aging effects of mulberry fruit extracts: In vitro and in vivo evaluations of the anti-glycation and antioxidant activities. J. Funct. Foods.

[5] Vo T.P., Pham T.V., Weina K., Tran T.N.H., Vo L.V., Nguyen P.T., Bui T.L.H., Phan T.H., Nguyen D.Q. (2023). Green extraction of phenolics and flavonoids from black mulberry fruit using natural deep eutectic solvents: Optimization and surface morphology. BMC Chem..

[6] Chen H., Yu W.S., Chen G., Meng S., Xiang Z.H., He N.J. (2018). Antinociceptive and Antibacterial Properties of Anthocyanins and Flavonols from Fruits of Black and Non-Black Mulberries. Molecules.

[7] Yu C., Hu X.M., Deng W., Li Y., Han G.M., Ye C.H. (2016). Soil fungal community comparison of different mulberry genotypes and the relationship with mulberry fruit sclerotiniosis. Sci. Rep..

[8] Fan W., Liu S.M., Xu Y.Z., Liu C.Y., Zhu P.P., Zhang S., Xia Z.Q., Zhao A.C. (2023). Stigma type and transcriptome analyses of mulberry revealed the key factors associated with Ciboria shiraiana resistance. Plant Physiol. Biochem..

[9] Yang M.X., Wang Y.H., Yang G.L., Wang Y.H., Liu F.Q., Chen C. (2024). A review of cumulative risk assessment of multiple pesticide residues in food: Current status, approaches and future perspectives. Trends Food Sci. Technol..

[10] van Rhijn N., Rhodes J. (2025). Evolution of antifungal resistance in the environment. Nat. Microbiol..

[11] Sabarwal A., Kumar K., Singh R.P. (2018). Hazardous effects of chemical pesticides on human health-Cancer and other associated disorders. Environ. Toxicol. Pharmacol..

[12] Bondareva L., Fedorova N. (2021). Pesticides: Behavior in Agricultural Soil and Plants. Molecules.

[13] Gould F., Brown Z.S., Kuzma J. (2018). Wicked evolution: Can we address the sociobiological dilemma of pesticide resistance?. Science.

[14] Dong M., Lu J.Z., Zhang W.J., Chen J.K., Li B. (2006). Canada goldenrod (*Solidago canadensis*): An invasive alien weed rapidly spreading in China. Acta Phytotaxon. Sin..

[15] Abhilasha D., Quintana N., Vivanco J., Joshi J. (2008). Do allelopathic compounds in invasive *Solidago canadensis* s.l. restrain the native European flora?. J. Ecol..

[16] Yuan Y.G., Wang B., Zhang S.S., Tang J.J., Tu C., Hu S.J., Yong J.W.H., Chen X. (2013). Enhanced allelopathy and competitive ability of invasive plant *Solidago canadensis* in its introduced range. J. Plant Ecol..

[17] Zhang S.S., Jin Y.L., Tang J.J., Chen X. (2009). The invasive plant *Solidago canadensis* L. suppresses local soil pathogens through allelopathy. Appl. Soil Ecol..

[18] Poljuha D., Sladonja B., Bozac M.U., Sola I., Damijanic D., Weber T. (2024). The Invasive Alien Plant *Solidago canadensis*: Phytochemical Composition, Ecosystem Service Potential, and Application in Bioeconomy. Plants.

[19] Anzlovar S., Janes D., Koce J.D. (2020). The Effect of Extracts and Essential Oil from Invasive *Solidago* spp. and Fallopia japonica on Crop-Borne Fungi and Wheat Germination. Food Technol. Biotechnol..

[20] Liu N., Song M.N., Sun Y.L., Yang F.Y., Yu H.N., Wu C., Sun Y.L., Chang W.Q., Ge D., Zhang H. (2021). Antifungal and Allelopathic Activities of Sesquiterpenes from *Solidago canadensis*. Curr. Org. Chem..

[21] Anzlovar S., Koce J.D. (2014). Antibacterial and Antifungal Activity of Aqueous and Organic Extracts from Indigenous and Invasive Species of Goldenrod (*Solidago* spp.) Grown in Slovenia. Phyton-Ann. Rei Bot..

[22] Apati P., Szentmihályi K., Balázs A., Baumann D., Hamburger M., Kristó T.S., Szőke É., Kéry Á. (2002). HPLC Analysis of the flavonoids in pharmaceutical preparations from canadian goldenrod (*Solidago canadensis*). Chromatographia.

[23] Uzelac Božac M., Poljuha D., Dudaš S., Bilić J., Šola I., Mikulič-Petkovšek M., Sladonja B. (2024). Phenolic Profile and Antioxidant Capacity of Invasive *Solidago canadensis* L.: Potential Applications in Phytopharmacy. Plants.

[24] Purmalis O., Klavins L., Niedrite E., Mezulis M., Klavins M. (2025). Invasive Plants as a Source of Polyphenols with High Radical Scavenging Activity. Plants.

[25] Gala-Czekaj D., Dziurka M., Bocianowski J., Synowiec A. (2021). Autoallelopathic potential of aqueous extracts from Canadian goldenrod (*Solidago canadensis* L.) and giant goldenrod (*S. gigantea* Aiton). Acta Physiol. Plant..

[26] Paulino B.N., Silva G.N.S., Araújo F.F., Néri-Numa I.A., Pastore G.M., Bicas J.L., Molina G. (2022). Beyond natural aromas: The bioactive and technological potential of monoterpenes. Trends Food Sci. Technol..

[27] Patrignani F., Siroli L., Serrazanetti D.I., Gardini F., Lanciotti R. (2015). Innovative strategies based on the use of essential oils and their components to improve safety, shelf-life and quality of minimally processed fruits and vegetables. Trends Food Sci. Technol..

[28] Ma Q., Xu Y.Q., Xiao H., Mariga A.M., Chen Y.P., Zhang X.C., Wang L., Li D., Li L., Luo Z.S. (2022). Rethinking of botanical volatile organic compounds applied in food preservation: Challenges in acquisition, application, microbial inhibition and stimulation. Trends Food Sci. Technol..

[29] Pavela R., Benelli G. (2016). Essential Oils as Ecofriendly Biopesticides? Challenges and Constraints. Trends Plant Sci..

[30] Střelková T., Jurkaninová L., Bušinová A., Nový P., Klouček P. (2024). Essential oils in vapour phase as antifungal agents in the cereal processing chain. Trends Food Sci. Technol..

[31] Senthil-Nathan S. (2020). A Review of Resistance Mechanisms of Synthetic Insecticides and Botanicals, Phytochemicals, and Essential Oils as Alternative Larvicidal Agents Against Mosquitoes. Front. Physiol..

[32] Liu S.M., Shao X.F., Wei Y.Z., Li Y.H., Xu F., Wang H.F. (2016). *Solidago canadensis* L. Essential Oil Vapor Effectively Inhibits Botrytis cinerea Growth and Preserves Postharvest Quality of Strawberry as a Food Model System. Front. Microbiol..

[33] Elshafie H.S., Grul’ová D., Baranová B., Caputo L., De Martino L., Sedlák V., Camele I., De Feo V. (2019). Antimicrobial Activity and Chemical Composition of Essential Oil Extracted from *Solidago canadensis* L. Growing Wild in Slovakia. Molecules.

[34] Wang C.Y., Jiang K., Liu J., Zhou J.W., Wu B.D. (2018). Moderate and heavy *Solidago canadensis* L. invasion are associated with decreased taxonomic diversity but increased functional diversity of plant communities in East China. Ecol. Eng..

[35] Wang S., Hafeez A., Zhang T.T., Rao M.J., Li S.C., Cai K.Z. (2025). Silicon-modified *Solidago canadensis* L. biochar suppresses soilborne disease and improves soil quality. Biochar.

[36] Kalemba D., Góra J., Kurowska A. (2007). Analysis of the Essential Oil of *Solidago canadensis*. Planta Medica.

[37] Huang B., Lei Y., Qin L., Liu J. (2012). Chemical Composition and Cytotoxic Activities of the Essential Oil from the Inflorescences of *Solidago canadensis* L., an Invasive Weed in Southeastern China. J. Essent. Oil Bear. Plants.

[38] El-Sherei M., Khaleel A., Motaal A.A., Abd-Elbaki P. (2014). Effect of Seasonal Variation on the Composition of the Essential Oil of *Solidago canadensis* Cultivated in Egypt. *J. Essent. Oil Bear*. Plants.

[39] Oh S.M., Kim D.Y., Lee S.Y., Song H.E., Kim I.S., Seo W.D., Lee J.H., Oh S.R., Lee D.Y., Ryu H.W. (2023). Comparisons of phenolic compounds and antioxidant activities during different growth stages in Artemisia gmelinii Weber ex Stechm with UPLC-QTOF/MS based on a metabolomics approach. Ind. Crops Prod..

[40] Sun P.Z., Lin S.Y., Li X.R., Li D.M. (2024). Different stages of flavor variations among canned Antarctic krill (*Euphausia superba*): Based on GC-IMS and PLS-DA. Food Chem..

[41] Han J., Hu Q., Wang Y. (2025). Rapid and accurate identification of Dendrobium species using FT-IR, FT-NIR, and data fusion with machine learning. Ind. Crops Prod..

[42] Hao Y.P., Kang J.M., Yang R., Li H., Cui H.X., Bai H.T., Tsitsilin A., Li J.Y., Shi L. (2022). Multidimensional exploration of essential oils generated via eight oregano cultivars: Compositions, chemodiversities, and antibacterial capacities. Food Chem..

[43] Das S., Singh V.K., Dwivedy A.K., Chaudhari A.K., Dubey N.K. (2021). Exploration of some potential bioactive essential oil components as green food preservative. LWT-Food Sci. Technol..

[44] Salehi B., Upadhyay S., Erdogan Orhan I., Kumar Jugran A., Jayaweera S.L.D., Dias D.A., Sharopov F., Taheri Y., Martins N., Baghalpour N. (2019). Therapeutic Potential of α- and β-Pinene: A Miracle Gift of Nature. Biomolecules.

[45] Leite-Andrade M.C., Neto L.N.D., Buonafina-Paz M.D.S., dos Santos F.D.G., Alves A.I.D., de Castro M.C.A.B., Mori E., de Lacerda B.C.G.V., Araújo I.M., Coutinho H.D.M. (2022). Antifungal Effect and Inhibition of the Virulence Mechanism of D-Limonene against Candida parapsilosis. Molecules.

[46] Wang X., Sun J.Y., Zhao S.H., Zhang F., Meng X.H., Liu B.J. (2023). Highly stable nanostructured lipid carriers containing candelilla wax for D-limonene encapsulation: Preparation, characterization and antifungal activity. Food Hydrocoll..

[47] Yu H., Lin Z.X., Xiang W.L., Huang M., Tang J., Lu Y., Zhao Q.H., Zhang Q., Rao Y., Liu L. (2022). Antifungal activity and mechanism of d-limonene against foodborne opportunistic pathogen *Candida tropicalis*. LWT-Food Sci. Technol..

[48] Judžentienė A. (2025). Compositional Variability of Essential Oils and Their Bioactivity in Native and Invasive Erigeron Species. Molecules.

[49] Iacovelli F., Romeo A., Lattanzio P., Ammendola S., Battistoni A., La Frazia S., Vindigni G., Unida V., Biocca S., Gaziano R. (2023). Deciphering the Broad Antimicrobial Activity of Melaleuca alternifolia Tea Tree Oil by Combining Experimental and Computational Investigations. Int. J. Mol. Sci..

[50] Połeć K., Broniatowski M., Wydro P., Hąc-Wydro K. (2020). The impact of β-myrcene—The main component of the hop essential oil—On the lipid films. J. Mol. Liq..

[51] Kohzaki K., Gomi K., Yamasaki-Kokudo Y., Ozawa R., Takabayashi J., Akimitsu K. (2009). Characterization of a sabinene synthase gene from rough lemon (*Citrus jambhiri*). J. Plant Physiol..

[52] Xu Z., Wu B., Wu C.H., Chen Q., Niu Y.F., Shi Z.J., Liang K., Rao X.P. (2025). Acrylpimaric acid-modified chitosan derivatives as potential antifungal agents against. Carbohydr. Polym..

[53] Zhao W.B., Zhao Z.M., Ma Y., Li A.P., Zhang Z.J., Hu Y.M., Zhou Y., Wang R., Luo X.F., Zhang B.Q. (2022). Antifungal activity and preliminary mechanism of pristimerin against Sclerotinia sclerotiorum. Ind. Crops Prod..

[54] Wu T.L., Zhang B.Q., Luo X.F., Li A.P., Zhang S.Y., An J.X., Zhang Z.J., Liu Y.Q. (2023). Antifungal efficacy of sixty essential oils and mechanism of oregano essential oil against Rhizoctonia solani. Ind. Crops Prod..

[55] Xu L., Xu X., Mao Y., Xu Y., Huang M. (2025). Characterization, vapor release behavior, vapor bio-functional performance and application of UV-responded modified polyvinyl alcohol bio-active films loaded with oregano essential oil microcapsules. Food Packag. Shelf Life.

[56] Huang Y.Z., Wang H.X., Wu C.N., Bao L.J., Su C., Qian Y.H. (2022). The effectiveness of Star Anise and Clove Essential Oils against Mulberry Sclerotia and Field Efficacy Test of Their Preparations. North Seric..

[57] Liu X.X., Wang Y., Zeng Z.B., Li D.B., Zhou L., Wang H.J., Lan F.J., Liu X.F. (2022). Screening of Eco-Friendly Agents for the Control of Mulberry Sclerotinia Diseases. China Seric..

[58] Grazzini A., Cavanaugh A.M. (2024). Fungal microtubule organizing centers are evolutionarily unstable structures. Fungal Genet. Biol..

[59] Steinberg G. (2007). Tracks for traffic: Microtubules in the plant pathogen Ustilago maydis. New Phytol..

[60] Jiang J., He K., Wang X.Y., Zhang Y., Guo X.H., Qian L., Gao X.H., Liu S.M. (2024). Transcriptional dynamics of Fusarium pseudograminearum under high fungicide stress and the important role of FpZRA1 in fungal pathogenicity and DON toxin production. Int. J. Biol. Macromol..

[61] Wang C., Li P., Cong W., Ma N., Zhou M., Hou Y. (2025). The transcription factor FgCreA modulates ergosterol biosynthesis and sensitivity to DMI fungicides by regulating transcription of FgCyp51A and FgErg6A in *Fusarium graminearum*. Int. J. Biol. Macromol..

[62] Xiao K.Q., Li Y.L., Gu S.Y., Liu L., Zhang G.P., Zhang Y.H., Zhang X.H., Liu J.L., Pan H.Y. (2025). Transcription Repressor SsGATA2 Regulates Broad-Spectrum Resistance to Fungicides and Pathogenicity in Sclerotinia sclerotiorum. J. Agric. Food Chem..

[63] Li K., Lv Y., Wu R.Z., Yu Z.W., Liang Y.L., Yu Z.C., Liang R.B., Tang L.F., Chen H.Y., Fan Z.J. (2024). Fungicidal Activity of Novel 6-Isothiazol-5-ylpyrimidin-4-amine-Containing Compounds Targeting Complex I Reduced Nicotinamide Adenine Dinucleotide Oxidoreductase. J. Agric. Food Chem..

[64] Oide S., Tanaka Y., Watanabe A., Inui M. (2019). Carbohydrate-binding property of a cell wall integrity and stress response component (WSC) domain of an alcohol oxidase from the rice blast pathogen Pyricularia oryzae. Enzym. Microb. Technol..

[65] John E., Chau M.Q., Hoang C.V., Chandrasekharan N., Bhaskar C., Ma L.S. (2024). Fungal Cell Wall-Associated Effectors: Sensing, Integration, Suppression, and Protection. Mol. Plant-Microbe Interact..

[66] Huang D.D., Qi H.T., Liu H., Yuan F.H., Yang C., Liu T. (2025). Two Birds with One Stone: Eco-Friendly Nano-Formulation Endows a Commercial Fungicide with Excellent Insecticidal Activity. Adv. Funct. Mater..

[67] Deng Q., Li Y., He W., Chen T., Liu N., Ma L., Qiu Z., Shang Z., Wang Z. (2025). A polyene macrolide targeting phospholipids in the fungal cell membrane. Nature.

[68] Ma C.X., Li Q.Q., Jia W.L., Shang H.P., Zhao J., Hao Y., Li C.Y., Tomko M., Zuverza-Mena N., Elmer W. (2021). Role of Nanoscale Hydroxyapatite in Disease Suppression of Fusarium-Infected Tomato. Environ. Sci. Technol..

[69] Ma Y.Z., Gao K., Yu H.H., Liu W.X., Qin Y.K., Xing R.E., Liu S., Li P.C. (2021). C-coordinated O-carboxymethyl chitosan Cu(II) complex exerts antifungal activity by disrupting the cell membrane integrity of *Phytophthora capsici* Leonian. Carbohydr. Polym..

[70] Yang L.P., Chen H.Y., Du P.R., Miao X.R., Huang S.Q., Cheng D.M., Xu H.H., Zhang Z.X. (2024). Inhibition mechanism of *Rhizoctonia solani* by pectin-coated iron metal-organic framework nanoparticles and evidence of an induced defense response in rice. J. Hazard. Mater..

[71] Hossain M.J., Romanov K.A., Jian J.F., Swaby L.C., Bandyopadhyay S., Guan I., Thomas S.M., Olive A.J., O’Connor T.J. (2025). *Bacterial pathogens* hijack host cell peroxisomes for replication vacuole expansion and integrity. Sci. Adv..

[72] Guo X.P., Li H.Y., Li Z.H., Cui Z.Q., Ma G.M., Nassor A.K., Guan Y., Pan X.H. (2025). Multi-stimuli-responsive pectin-coated dendritic mesoporous silica nanoparticles with Eugenol as a sustained release nanocarrier for the control of tomato bacterial wilt. J. Nanobio.

[73] Zhang W., Niu Q., Cui Y., Fan K., Wang X. (2025). Copper-Doped Prussian Blue Nanozymes: Targeted Starvation Therapy Against Gram-Positive Bacteria via the ABC Transporter Inhibition. Adv. Funct. Mater..

[74] Ding M.N., Zhang Q., Shi F.Y., Zhou L.P., Guo X.C., Li Q.Y., Luo L.L., Miao Y.C., Huo Y.N. (2025). Initiative invasion promoted photoelectrocatalytic antibacterial function on ZIF-67@CoO@Co foil photoanode. Chem. Eng. J..

[75] Tong Y.Q., Jiang W., Li J.Y., Teng P., Wei L.L., Xu H.E., Jiang X.Q., Hu Y. (2025). Biomimetic nanodrug breaching tumor cell membrane barrier for high-efficiency drug delivery. Chem. Eng. J..

[76] Liu X.L., Li H., Qi G.B., Qian Y.Y., Li B.W., Shi L.L., Liu B. (2024). Combating Fungal Infections and Resistance with a Dual-Mechanism Luminogen to Disrupt Membrane Integrity and Induce DNA Damage. J. Am. Chem. Soc..

[77] Zhang T.Y., Chen Y.Q., Tan J.C., Zhou J.A., Chen W.N., Jiang T., Zha J.Y., Zeng X.K., Li B.W., Wei L.Q. (2024). Global fungal-host interactome mapping identifies host targets of candidalysin. Nat. Commun..

[78] Jaramillo-Colorado B.E., Arroyo-Salgado B., Palacio-Herrera F.M. (2025). Antifungal activity of four *Piper* genus essential oils against postharvest fungal *Fusarium* spp. isolated from *Mangifera indica* L. and *Persea americana* Mill. fruits. Ind. Crops Prod..

[79] Nkuimi Wandjou J.G., Quassinti L., Gudžinskas Z., Nagy D.U., Cianfaglione K., Bramucci M., Maggi F.J.C. (2020). Chemical composition and antiproliferative effect of essential oils of four Solidago species (*S. canadensis*, *S. gigantea*, *S. virgaurea* and *S.× niederederi*). Chem. Biodivers..

[80] Zhao Z., Yu M., Wei Y., Xu F., Jiang S., Chen Y., Ding P., Shao X. (2025). Cinnamon essential oil causes cell membrane rupture and oxidative damage of *Rhizopus stolonifer* to control soft rot of peaches. Food Control.

