# Supplementary material for: Multivariate Statistical Analysis of Solidago canadensis L. Essential Oil and Its Antifungal Mechanism Against Mulberry Sclerotinia Diseases
Source: Int J Mol Sci. 2025 Dec 20;27(1):49. doi: 10.3390/ijms27010049 (PMC12785313; doi:10.3390/ijms27010049)
Supplement: Supplementary file 1 [file ijms-27-00049-s001.zip › ijms-4010442-supplementary.pdf]

Supplementary Materials

Multivariate Statistical Analysis of *Solidago canadensis* L. Essential Oil and its Antifungal Mechanism Against Mulberry Sclerotinia Diseases

Jia-Xin Yang [1,2], Zhen-Zhen Lu [1,2], Sen Chen [1,2], Shi-Yi Lin [1,2], Xiao-Hui Yao [1,2], Tao Chen [1,2,*], Dong-Yang Zhang [1,2,*]

[1] Jiangsu Key Laboratory of Sericultural and Animal Biotechnology, School of Biotechnology, Jiangsu University of Science and Technology, Zhenjiang 212100, China

[2] Key Laboratory of Silkworm and Mulberry Genetic Improvement, Ministry of Agriculture and Rural Affairs, Sericultural Scientific Research Center, Chinese Academy of Agricultural Sciences, Zhenjiang 212100, China

* Correspondence: chentao@just.edu.cn (T.C.)

zhangdongyang1987@just.edu.cn (D.-Y.Z.)

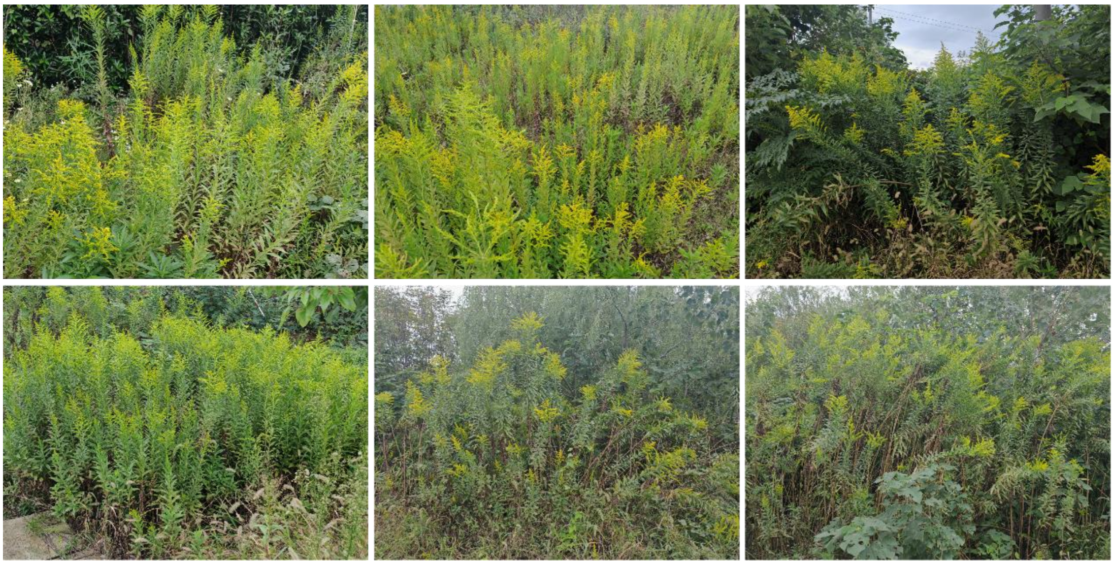

Figure S1. Photos of *S. canadensis* L. taken at the sampling site.

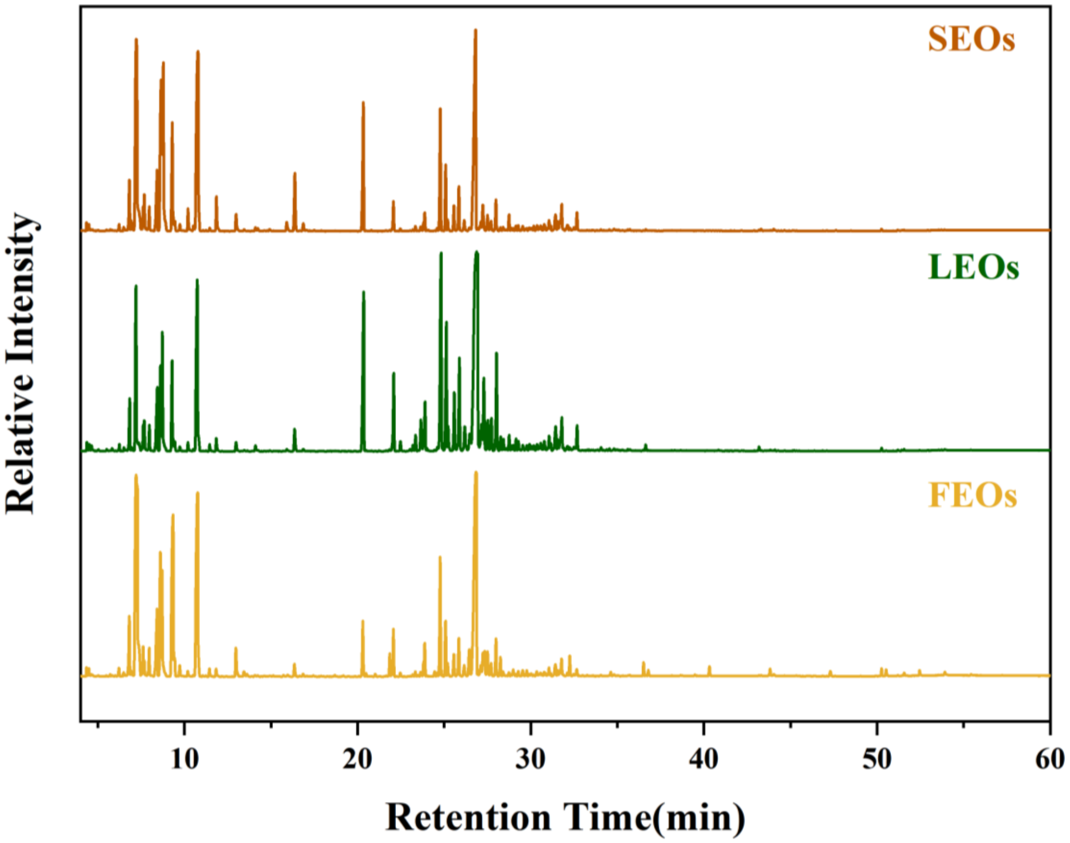

Figure S2. GC-MS chromatograms of SEOs, LEOs, and FEOs.

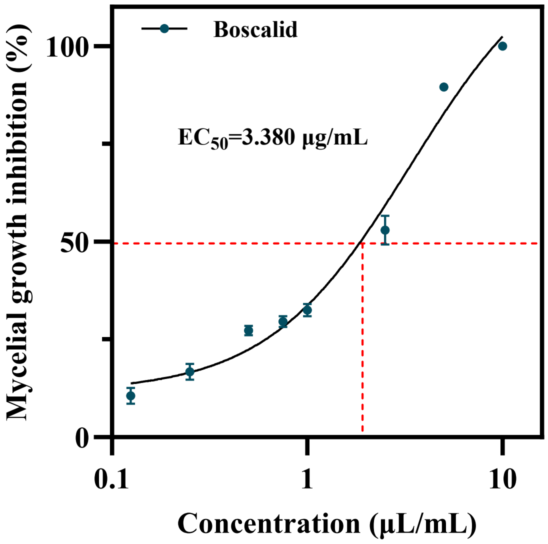

Figure S3. EC_{50} of positive control (boscalid).

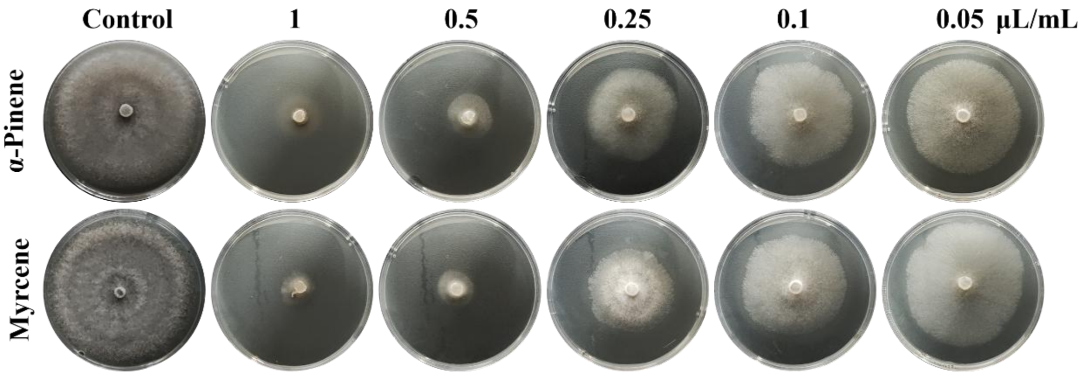

Figure S4. Inhibitory activity of mycelial growth of α-pinene and myrcene on PDA medium.

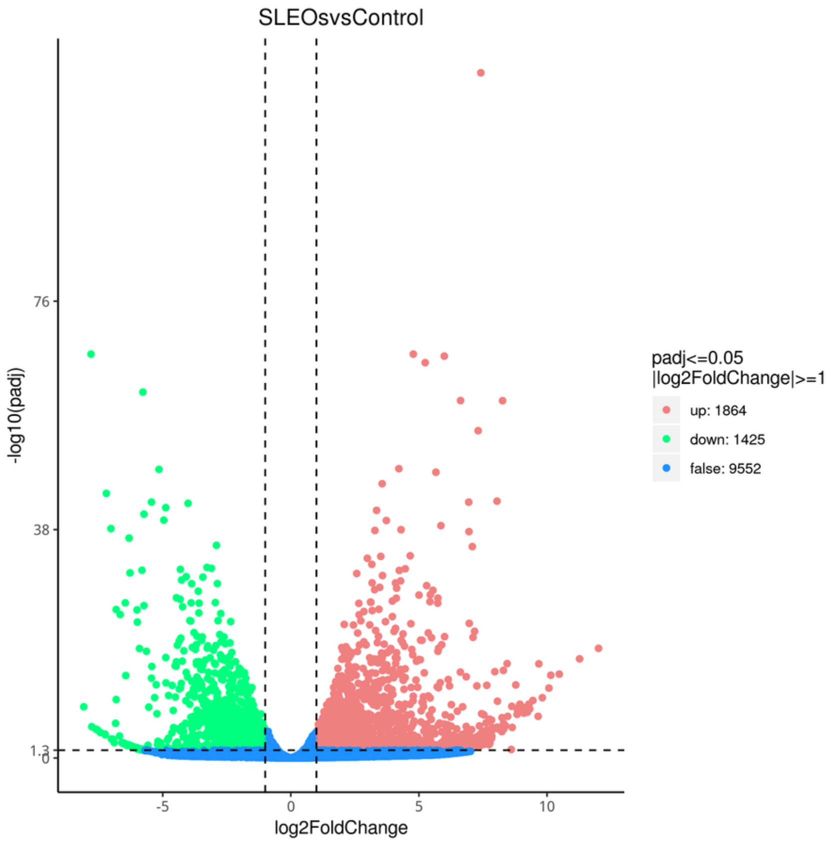

Figure S5. The number of DEGs of *C. shiraiana* after treatment with FEOs.

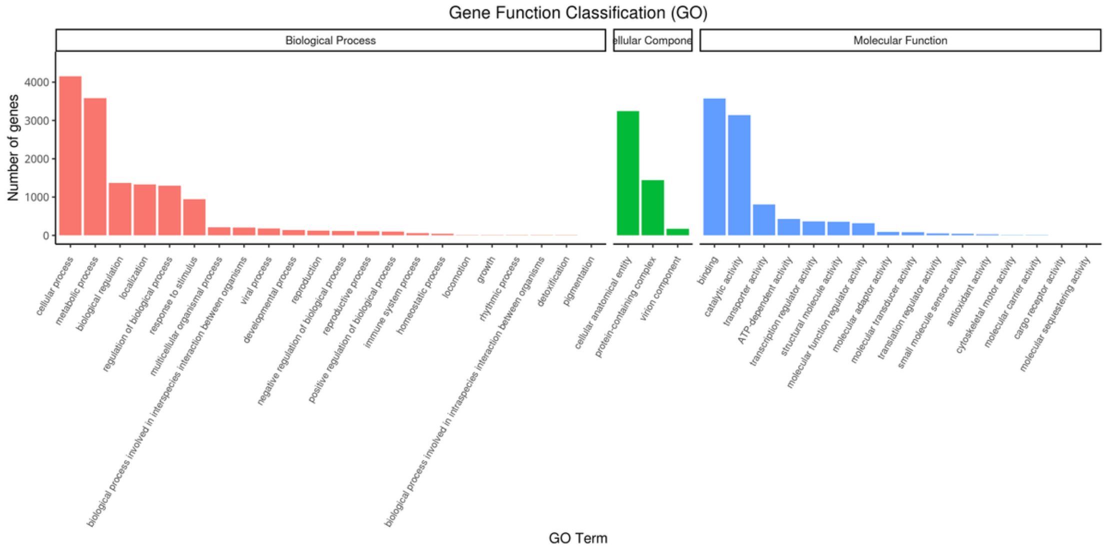

Figure S6. Gene function classification based on GO.

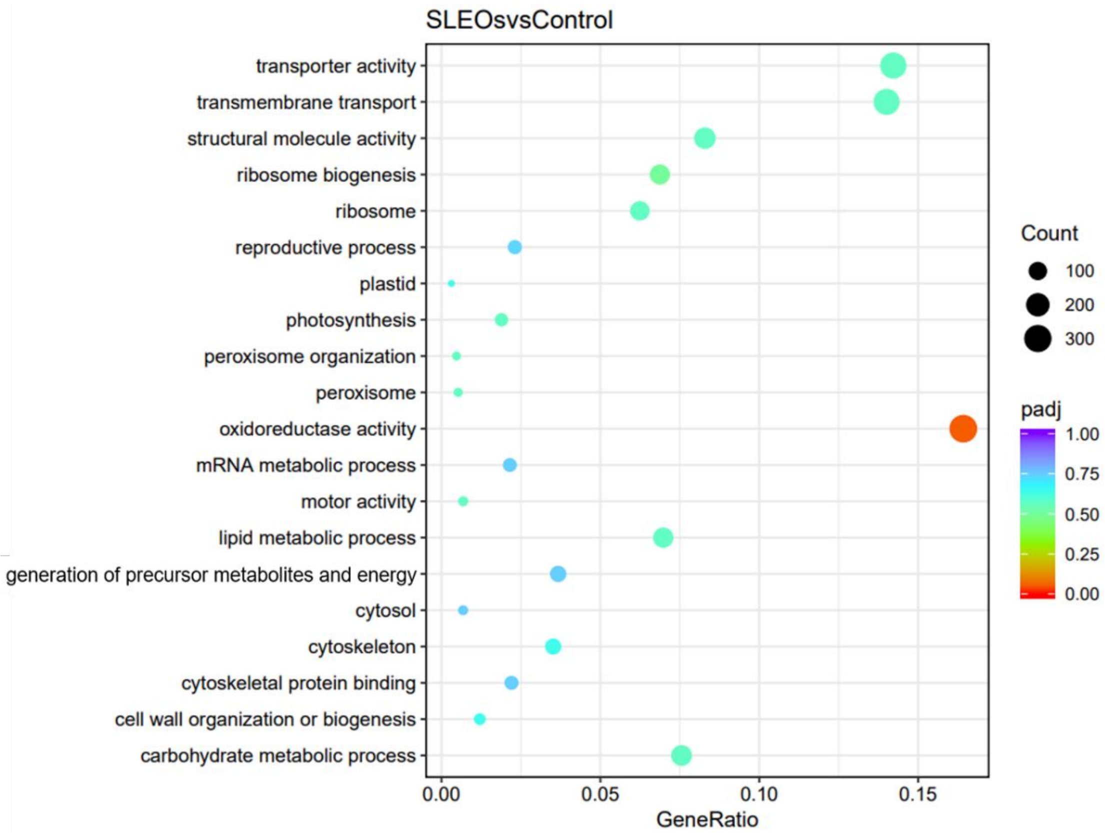

Figure S7. GO enrichment analysis of DEGs in *C. shiraiana.*

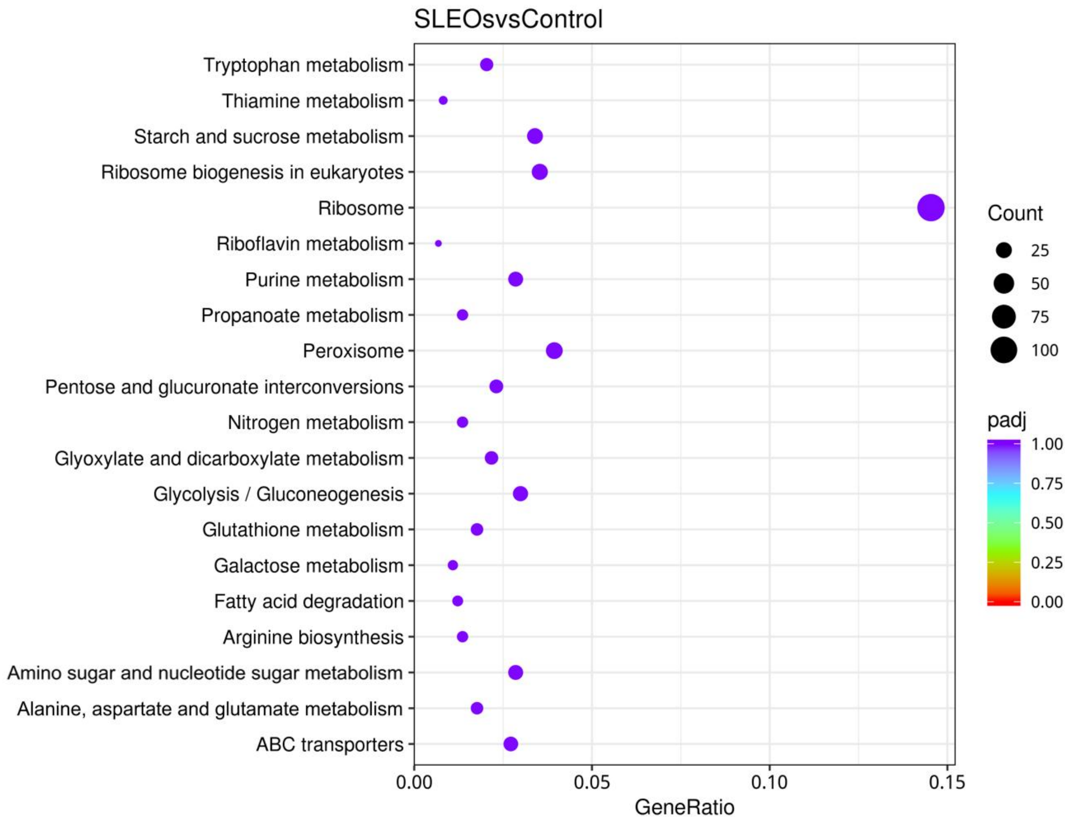

Figure S8. KEGG enrichment analysis of DEGs in *C. shiraiana.*

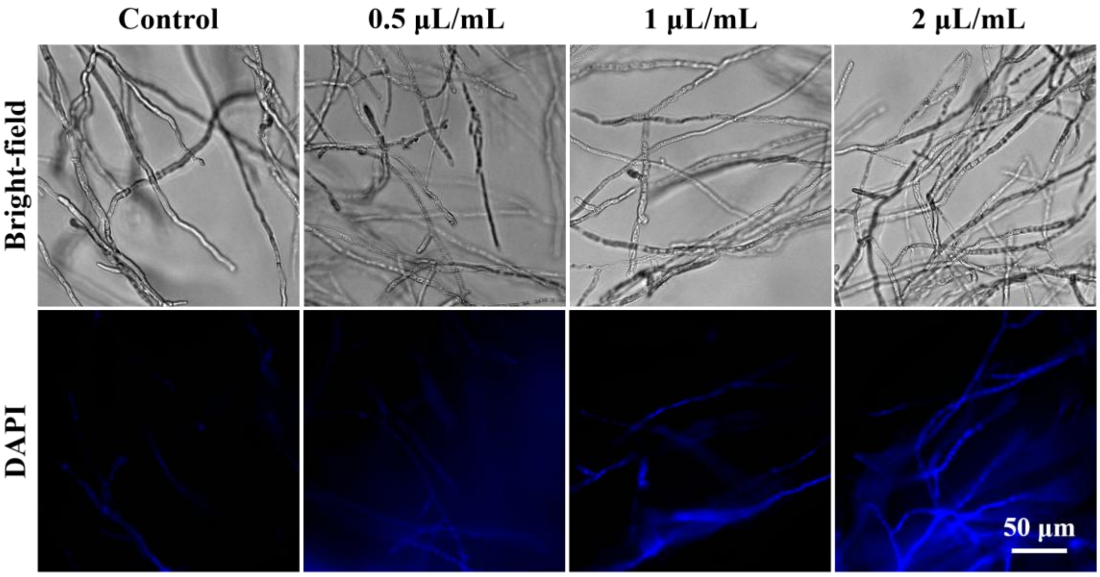

Figure S9. DAPI staining of *C. shiraiana.*

Table S1

Chemical compositions of EOs derived from flower, leaf and stem of *S. canadensis L.*

	Compounds	CAS	Molecular formula	RI	Concentration (%)			Category
					Flowers	Leaves	Stems	
1	α-Thujene	2867-05-2	$C_{10}H_{16}$	929	0.16±0.00[b]	0.11±0.00[c]	0.29±0.01[a]	Monoterpene
2	α-Pinene	80-56-8	$C_{10}H_{16}$	937	18.04±0.11[a]	5.20±0.16[c]	13.82±0.40[b]	Monoterpene
3	Camphene	79-92-5	$C_{10}H_{16}$	952	0.31±0.00[c]	0.62±0.00[b]	0.91±0.08[a]	Monoterpene
4	Artemisia ketone	546-49-6	$C_{10}H_{16}O$	1062	2.51±0.18[a]	2.25±0.08[b]	2.45±0.02[ab]	Oxygenated monoterpenes
5	Sabinene	3387-41-5	$C_{10}H_{16}$	974	3.86±0.19[b]	2.29±0.05[c]	8.15±0.05[a]	Monoterpene
6	β-Pinene	127-91-3	$C_{10}H_{16}$	979	3.52±0.06[c]	3.68±0.02[b]	8.39±0.10[a]	Monoterpene
7	Myrcene	123-35-3	$C_{10}H_{16}$	991	9.66±0.14[a]	2.97±0.01[c]	4.25±0.02[b]	Monoterpene
8	α-Phellandrene	99-83-2	$C_{10}H_{16}$	1005	0.30±0.00[a]	0.11±0.01[c]	0.21±0.00[b]	Monoterpene
9	α-Terpinene	99-86-5	$C_{10}H_{16}O$	1017	0.18±0.01[c]	0.26±0.01[b]	0.76±0.01[a]	Oxygenated monoterpenes
10	p-Cymene	99-87-6	$C_{10}H_{14}$	1025	-	-	0.20±0.00	Monoterpene
11	Limonene	5989-27-5	$C_{10}H_{16}$	1031	12.86±0.27[b]	8.11±0.02[c]	13.55±0.05[a]	Monoterpene
12	(Z)-β-Ocimene	3338-55-4	$C_{10}H_{16}$	1038	0.27±0.01[a]	0.21±0.00[b]	0.11±0.00[c]	Monoterpene
13	γ-Terpinene	99-85-4	$C_{10}H_{16}$	1060	0.28±0.02[c]	0.42±0.01[b]	1.22±0.01[a]	Monoterpene
14	Terpinolene	586-62-9	$C_{10}H_{16}$	1088	1.08±0.04[a]	0.31±0.00[c]	0.66±0.02[b]	Monoterpene
15	Linalool	78-70-6	$C_{10}H_{18}O$	1099	0.21±0.01	-	0.10±0.00	Oxygenated monoterpenes
16	Nonanal	124-19-6	$C_9H_{18}O$	1274	0.11±0.00	-	-	Aldehyde
17	(E)-4,8-Dimethyl-1,3,7-nonatriene	19945-61-0	$C_{11}H_{18}$	1116	-	0.20±0.01	0.13±0.00	Others
18	(E)-p-2-Menthen-1-ol	29803-81-4	$C_{10}H_{18}O$	1140	-	-	0.12±0.00	Oxygenated monoterpenes
19	(Z)-2-p-Menthen-1-ol	29803-82-5	$C_{10}H_{18}O$	1122	-	-	0.09±0.00	Oxygenated monoterpenes
20	Borneol	507-70-0	$C_{10}H_{18}O$	1167	-	0.10±0.00	0.43±0.02	Oxygenated monoterpenes

(continued on next page)

Table S1 (*continued*)

	Compounds	CAS	Molecular formula	RI	Concentration (%)			Category
21	Terpinen-4-ol	562-74-3	$C_{10}H_{18}O$	1177	0.47±0.02[c]	0.79±0.02[b]	2.35±0.02[a]	Oxygenated monoterpenes
22	α-Terpineol	98-55-5	$C_{10}H_{18}O$	1189	0.10±0.01[b]	0.08±0.00[c]	0.29±0.00[a]	Oxygenated monoterpenes
23	Bornyl acetate	76-49-3	$C_{12}H_{20}O_2$	1285	1.76±0.08[c]	6.54±0.10[a]	5.20±0.07[b]	Ester
24	Lavandulyl acetate	20777-39-3	$C_{12}H_{20}O_2$	1289	0.09±0.00	-	-	Ester
25	δ-EIemene	20307-84-0	$C_{15}H_{24}$	1338	1.52±0.08[b]	2.45±0.04[a]	0.96±0.01[c]	Sesquiterpene
26	α-Cubebene	17699-14-8	$C_{15}H_{24}$	1351	0.13±0.01	0.30±0.01	-	Sesquiterpene
27	α-Ylangene	14912-44-8	$C_{15}H_{24}$	1372	0.06±0.00	0.18±0.01	-	Sesquiterpene
28	α-Copaene	3856-25-5	$C_{15}H_{24}$	1376	0.15±0.01[b]	0.46±0.01[a]	0.15±0.00[b]	Sesquiterpene
29	β-Chamigrene	18431-82-8	$C_{15}H_{24}$	1476	0.05±0.00	-	-	Sesquiterpene
30	β-Bourbonene	5208-59-3	$C_{15}H_{24}$	1384	0.15±0.01[b]	0.97±0.01[a]	0.14±0.00[b]	Sesquiterpene
31	β-Copaene	18252-44-3	$C_{15}H_{24}$	1432	2.92±0.06[b]	4.52±0.04[a]	2.11±0.04[c]	Sesquiterpene
32	β-Elemene	515-13-9	$C_{15}H_{24}$	1391	0.98±0.03[b]	1.43±0.02[a]	0.54±0.01[c]	Sesquiterpene
33	α-Gurjunene	489-40-7	$C_{15}H_{24}$	1409	0.15±0.00	-	-	Sesquiterpene
34	β-Ylangene	20479-06-5	$C_{15}H_{24}$	1421	5.23±0.04[b]	8.37±0.09[a]	4.11±0.08[c]	Sesquiterpene
35	γ-Elemene	29873-99-2	$C_{15}H_{24}$	1433	0.46±0.01[b]	0.71±0.04[a]	0.29±0.00[c]	Sesquiterpene
36	Isogermacrene D	317819-80-0	$C_{15}H_{24}$	1448	1.12±0.01[b]	1.86±0.02[a]	0.82±0.02[c]	Sesquiterpene
37	γ-Muurolene	30021-74-0	$C_{15}H_{24}$	1477	2.94±0.10[b]	5.23±0.17[a]	2.36±0.09[c]	Sesquiterpene
38	β-Cubebene	13744-15-5	$C_{15}H_{24}$	1390	0.58±0.01[b]	0.91±0.04[a]	0.40±0.01[c]	Sesquiterpene
39	γ-Gurjunene	22567-17-5	$C_{15}H_{24}$	1473	0.96±0.00[a]	0.51±0.01[b]	0.13±0.00[c]	Sesquiterpene
40	Germacrene D	23986-74-5	$C_{15}H_{24}$	1481	18.52±0.49[b]	24.23±0.19[a]	15.15±0.23[c]	Sesquiterpene
41	Eremophilene	10219-75-7	$C_{15}H_{24}$	1494	0.06±0.00[b]	0.10±0.00[a]	0.04±0.00[c]	Sesquiterpene

(continued on next page)

Table S1 (*continued*)

	Compounds	CAS	Molecular formula	RI	Concentration (%)			Category
42	Cubebol	23445-02-5	$C_{15}H_{26}O$	1515	0.33±0.02^{b}	0.71±0.05^{a}	0.37±0.01^{b}	Oxygenated sesquiterpenoids
43	β-Cadinene	523-47-7	$C_{15}H_{24}$	1518	0.80±0.02^{b}	0.91±0.01^{a}	0.48±0.01^{c}	Sesquiterpene
44	γ-Cadinene	39029-41-9	$C_{15}H_{24}$	1513	0.44±0.03^{b}	0.95±0.01^{a}	0.32±0.03^{c}	Sesquiterpene
45	δ-Cadinene	60305-17-1	$C_{15}H_{24}$	1522	1.17±0.08^{b}	3.18±0.04^{a}	0.99±0.01^{c}	Sesquiterpene
46	Nootkatene	5090-61-9	$C_{15}H_{22}$	1511	0.53±0.02^{a}	0.38±0.00^{b}	0.09±0.00^{c}	Sesquiterpene
47	α-Cadinene	24406-05-1	$C_{15}H_{22}$	1538	0.13±0.01^{b}	0.30±0.00^{a}	0.10±0.00^{c}	Sesquiterpene
48	Elemol	639-99-6	$C_{15}H_{26}O$	1549	0.10±0.00^{c}	0.39±0.01^{b}	0.45±0.00^{a}	Oxygenated sesquiterpenoids
49	Betulenol	50277-33-3	$C_{15}H_{24}O$	1686	0.04±0.00^{c}	0.12±0.00^{a}	0.06±0.00^{b}	Oxygenated sesquiterpenoids
50	Germacrene B	15423-57-1	$C_{15}H_{24}$	1557	0.13±0.02^{a}	0.14±0.01^{a}	0.06±0.00^{b}	Sesquiterpene
51	E-Nerolidol	40716-66-3	$C_{15}H_{26}O$	1564	0.05±0.00^{c}	0.29±0.01^{a}	0.13±0.00^{b}	Oxygenated sesquiterpenoids
52	1,5-Epoxysalvial-4 (14)-ene	88395-47-5	$C_{15}H_{24}O$	1573	0.16±0.03^{b}	0.25±0.00^{a}	0.14±0.00^{b}	Oxygenated sesquiterpenoids
53	(Z)-3,7-dimethylocta-2,6-dienyl 2-methylbutyrate	51117-19-2	$C_{15}H_{26}O_2$	1576	0.20±0.01^{a}	0.17±0.00^{b}	0.16±0.00^{b}	Oxygenated sesquiterpenoids
54	(E)-Isovalencenol	22387-74-2	$C_{15}H_{24}O$	1788	0.17±0.01	0.15±0.00	-	Oxygenated sesquiterpenoids
55	Isoaromadendrene epoxide	499134-59-7	$C_{15}H_{24}O$	1603	0.07±0.00^{c}	0.16±0.00^{a}	0.09±0.00^{b}	Oxygenated sesquiterpenoids
56	β-Acorenol	28400-11-5	$C_{15}H_{26}O$	1649	0.11±0.01^{b}	0.14±0.01^{a}	0.09±0.01^{c}	Oxygenated sesquiterpenoids
57	Guaiol	489-86-1	$C_{15}H_{26}O$	1596	0.03±0.00^{c}	0.11±0.00^{b}	0.12±0.00^{a}	Oxygenated sesquiterpenoids
58	Copaborneol	21966-93-8	$C_{15}H_{26}O$	1639	0.13±0.01^{b}	0.15±0.00^{a}	0.15±0.00^{a}	Oxygenated sesquiterpenoids
59	ent-Germacra-4 (15),5,10 (14)-trien-1β-ol	81968-62-9	$C_{15}H_{24}O$	1695	0.30±0.01^{c}	0.87±0.02^{a}	0.74±0.00^{b}	Oxygenated sesquiterpenoids
60	Junenol	472-07-1	$C_{15}H_{26}O$	1614	0.10±0.01^{b}	0.27±0.01^{a}	0.26±0.00^{a}	Oxygenated sesquiterpenoids

(continued on next page)

Table S1 (*continued*)

	Compounds	CAS	Molecular formula	RI	Concentration (%)			Category
61	Isospathulenol	88395-46-4	$C_{15}H_{24}O$	1638	0.24±0.01[c]	0.41±0.01[a]	0.30±0.00[b]	Oxygenated sesquiterpenoids
62	Cubenol	21284-22-0	$C_{15}H_{26}O$	1642	0.08±0.00[c]	0.20±0.00[a]	0.16±0.00[b]	Oxygenated sesquiterpenoids
63	α-epi-Muurolol	19912-62-0	$C_{15}H_{26}O$	1642	0.42±0.01[c]	0.88±0.02[a]	0.66±0.02[b]	Oxygenated sesquiterpenoids
64	T-cadinol	5937-11-1	$C_{15}H_{26}O$	1640	0.15±0.01[b]	0.23±0.02[a]	0.23±0.02[a]	Oxygenated sesquiterpenoids
65	β-Eudesmol	473-15-4	$C_{15}H_{26}O$	1649	0.60±0.03[c]	1.18±0.02[b]	1.25±0.05[a]	Oxygenated sesquiterpenoids
66	Bulnesol	22451-73-6	$C_{15}H_{26}O$	1667	0.13±0.01[b]	0.21±0.01[b]	0.31±0.00[a]	Oxygenated sesquiterpenoids
67	15-Hydroxyculmorin	144447-99-4	$C_{15}H_{26}O_3$	2143	0.09±0.00[a]	0.07±0.01[b]	0.10±0.00[a]	Oxygenated sesquiterpenoids
68	Cyperotundone	3466-15-7	$C_{15}H_{22}O$	1687	0.11±0.00	-	-	Oxygenated sesquiterpenoids
69	Neophytadiene	504-96-1	$C_{20}H_{38}$	1838	-	0.16±0.00	-	Others
70	Saussurea lactone	23527-07-3	$C_{15}H_{22}O_2$	1806	0.12±0.01[a]	0.09±0.00[b]	0.10±0.01[b]	Ester
71	Phytone	502-69-2	$C_{18}H_{36}O$	1844	0.18±0.01	-	-	Others
72	Phytol	150-86-7	$C_{20}H_{40}O$	2114	-	0.12±0.01	-	Others

Notes: RI, Retention indices; values are reported as mean ± standard deviation of three parallel experiments; '-' means not detected. Compound identification was based on the NIST 20 mass spectral database and RI values. Means with different letters in a row are statistically significant (p<0.05).
